# Caspase-1-Dependent and -Independent Cell Death Pathways in *Burkholderia pseudomallei* Infection of Macrophages

**DOI:** 10.1371/journal.ppat.1003986

**Published:** 2014-03-13

**Authors:** Antje Bast, Kathrin Krause, Imke H. E. Schmidt, Matsayapan Pudla, Stefanie Brakopp, Verena Hopf, Katrin Breitbach, Ivo Steinmetz

**Affiliations:** 1 Friedrich Loeffler Institute of Medical Microbiology, University Medicine Greifswald, Greifswald, Germany; 2 Department of Microbiology, Faculty of Science, Mahidol University, Bangkok, Thailand; Duke-National University of Singapore Graduate Medical School, Singapore

## Abstract

The cytosolic pathogen *Burkholderia pseudomallei* and causative agent of melioidosis has been shown to regulate IL-1β and IL-18 production through NOD-like receptor NLRP3 and pyroptosis via NLRC4. Downstream signalling pathways of those receptors and other cell death mechanisms induced during *B. pseudomallei* infection have not been addressed so far in detail. Furthermore, the role of *B. pseudomallei* factors in inflammasome activation is still ill defined. In the present study we show that caspase-1 processing and pyroptosis is exclusively dependent on NLRC4, but not on NLRP3 in the early phase of macrophage infection, whereas at later time points caspase-1 activation and cell death is NLRC4- independent. In the early phase we identified an activation pathway involving caspases-9, -7 and PARP downstream of NLRC4 and caspase-1. Analyses of caspase-1/11-deficient infected macrophages revealed a strong induction of apoptosis, which is dependent on activation of apoptotic initiator and effector caspases. The early activation pathway of caspase-1 in macrophages was markedly reduced or completely abolished after infection with a *B. pseudomallei* flagellin FliC or a T3SS3 BsaU mutant. Studies using cells transfected with the wild-type and mutated T3SS3 effector protein BopE indicated also a role of this protein in caspase-1 processing. A T3SS3 inner rod protein BsaK mutant failed to activate caspase-1, revealed higher intracellular counts, reduced cell death and IL-1β secretion during early but not during late macrophage infection compared to the wild-type. Intranasal infection of BALB/c mice with the BsaK mutant displayed a strongly decreased mortality, lower bacterial loads in organs, and reduced levels of IL-1β, myeloperoxidase and neutrophils in bronchoalveolar lavage fluid. In conclusion, our results indicate a major role for a functional T3SS3 in early NLRC4-mediated caspase-1 activation and pyroptosis and a contribution of late caspase-1-dependent and -independent cell death mechanisms in the pathogenesis of *B. pseudomallei* infection.

## Author Summary

Inflammasome activation is important for host defence against bacterial infection. Many gram-negative pathogens use secretion systems to inject bacterial proteins such as flagellin or structural components of the secretion machinery itself into the host cytosol leading to caspase-1 activation and pyroptotic cell death. However, little is known about the *B. pseudomallei* factors that trigger caspase-1 activation as well as downstream signalling pathways and effector mechanisms of caspase-1. Here, we identified the *B. pseudomallei* T3SS3 inner rod protein BsaK as an early activator of caspase-1-dependent cell death and IL-1β secretion in primary macrophages and as a virulence factor in murine melioidosis. We could show that upon infection of macrophages, caspase-7 is activated downstream of the NLRC4/caspase-1 inflammasome and requires caspase-9 processing. Although caspase-7 was essential for cleavage of the DNA damage sensor PARP during pyroptosis, it did neither contribute to cytokine production nor *B. pseudomallei* growth restriction by promoting early macrophage death. In addition to a rapid NLRC4/caspase-1- dependent induction of pyroptosis in wild-type macrophages, we observed a delayed activation of classical apoptosis in macrophages lacking caspase-1/11. Thus, initiation of different cell death pathways seems to be an effective strategy to limit intracellular *B. pseudomallei* infection.

## Introduction

The innate immune system provides the first line of defence against microbial infection. It is activated in response to invading microbes by the engagement of pattern-recognition receptors (PRRs) including membrane-bound Toll-like receptors (TLRs) and cytosolic nucleotide-binding oligomerization domain (NOD)-like receptors (NLRs). PRRs recognize microbial pathogen-associated molecular patterns (PAMPs) or endogenous damage-associated molecular patterns (DAMPs) leading to activation of host defence pathways that result in the clearance of infection. Some NLRs can initiate assembly of the inflammasome, a multiprotein complex that typically contains a NLR, the adapter molecule ASC (apoptosis-associated speck-like protein containing a caspase recruitment domain (CARD)) and the protease caspase-1 [1].

NLRP3 and NLRC4 are the most extensively-studied NLR molecules. The NLRP3 inflammasome is triggered by a wide variety of stimuli including environmental irritants and host-derived danger signals that are indicative of cellular damage or metabolic dysregulation (DAMPs). Multiple pathogens, PAMPs and other pathogen-associated molecules such as bacterial pore-forming toxins may activate the NLRP3 inflammasome [2], [3]. A number of gram-negative bacteria are thought to induce caspase-1 activation via the NLRC4 inflammasome including *Salmonella* [4], [5], *Legionella* [6], *Pseudomonas* [7]–[9], *Yersinia* [10] and *Burkholderia* [11]–[13]. NLRC4 specifically responds to a functional type III (T3SS) or type IV (T4SS) secretion system either indirectly by detecting flagellin [4]–[6], [8], [9], [14] or directly by detecting T3SS rod proteins through a sequence motif that is also found in flagellin FliC [11], [15]. The specificity of the NLRC4 inflammasome for different bacterial ligands is determined by its interaction with distinct members of the NAIP (neuronal apoptosis inhibitor protein) family. Murine NAIP5 and NAIP6 are required for sensing bacterial flagellin in the host cytoplasm, whereas murine NAIP2 specifically detects T3SS inner rod proteins such as PrgJ from *Salmonella enterica* subspecies Typhimurium (*S. typhimurium*) [13], [16]. However, human NAIP binds to T3SS needle subunits from several bacteria including *S. typhimurium* PrgI [13]. Moreover, the *S. typhimurium* T3SS effector protein SopE can activate Rho GTPases and thereby triggers host cell invasion and caspase-1 activation [17].

Caspase-1 cleaves the immature forms of IL-1β and IL-18 prior to their release as biologically active inflammatory cytokines. It also induces pyroptotic cell death, a key innate defence mechanism against intracellular bacteria [18], [19]. Pyroptosis occurs within minutes after inflammasome activation and is associated with immediate plasma membrane permeabilisation and release of cytoplasmic contents leading to inflammation. In contrast, apoptosis is considered as immunologically silent cell death as membrane integrity is maintained. Apoptotic initiator caspases-2, -8, and -9 induce cleavage of executioner caspases such as caspases-3, -6 and -7 cleaving target proteins to trigger host cell death. NLRC4-mediated activation of caspase-1 has previously been reported to result in proteolytic processing of caspase-7 during *S. typhimurium* [20] or *L. pneumophila* infection leading to restriction of *Legionella* replication in infected murine macrophages [21].

*Burkholderia pseudomallei* is a gram-negative flagellate bacterium that causes melioidosis, a disease endemic to Southeast Asia and northern Australia [22], [23]. Infection results from subcutaneous inoculation, inhalation or ingestion and may lead to a wide range of clinical manifestations including pneumonia, organ abscesses and septicaemia. *B. pseudomallei* can invade phagocytic and non-phagocytic cells, is able to escape from the endocytic vacuole and to replicate in the host cell cytosol by the use of a T3SS3 [23]. Macrophages and interferon-γ (IFN-γ) are critical for the early control of *B. pseudomallei* infection [24]–[27].

*B. pseudomallei* is capable of inducing caspase-1-dependent cell death in murine macrophages [28]. In a previous study we described that this rapid pyroptotic cell death is involved in defence against *B. pseudomallei* by removing the pathogen's replication niche but caspase-1 contributes to the control of intracellular bacterial replication in macrophages as well [29]. Work by Ceballos-Olvera et al. [12] revealed that *B. pseudomallei* can activate the caspase-1 inflammasome by both NLRC4 and NLRP3. NLRC4 mainly regulates pyroptosis, whereas NLRP3 is primarily important for production of IL-1β and IL-18. IL-1β can play a deleterious role in melioidosis by causing excessive neutrophil recruitment and tissue damage but IL-18 may be protective through induction of IFN-γ production. Furthermore, it has been shown that cytosolic flagellin from closely related but far less virulent *B. thailandensis* is not able to interact with NAIP5-NLRC4 and to stimulate caspase-1 activation, whereas the T3SS3 inner rod protein BsaK can interact with the NAIP2-NLRC4 inflammasome. Delivery of recombinant BsaK from *B. thailandensis* into macrophages stimulates NLRC4-dependent caspase-1 activation, pyroptosis and IL-1β release [13]. Using a retroviral lethality screen NLRC4 has been found to respond to recombinant BsaK from *B. pseudomallei* as well [11]. However, the contribution of BsaK in the context of a *B. pseudomallei* infection has not been addressed. A recent study further demonstrated that murine caspase-11 can contribute to pyroptotic cell death through an unknown non-canonical inflammasome in response to cytosolic bacteria including *B. pseudomallei* and *B. thailandensis* [30].

As host cell death is an effective strategy to limit *Burkholderia* infection, this study aimed to analyse the general features and the course of cell death in wild-type macrophages and in caspase-1/11 knockout macrophages unable to induce pyroptosis and to identify downstream signalling pathways of *Burkholderia*-induced caspase-1 activation. Finally, we sought to characterize the role of *B. pseudomallei* flagellin FliC, the T3SS3 effector protein BopE and the inner rod protein BsaK in caspase-1 activation, pyroptosis induction and IL-1β secretion as well as their function in primary macrophages and in murine melioidosis.

## Results

### *Burkholderia* infection leads to a strain-dependent activation of caspases-1, -9 and -7 in macrophages

**Figure 1:**
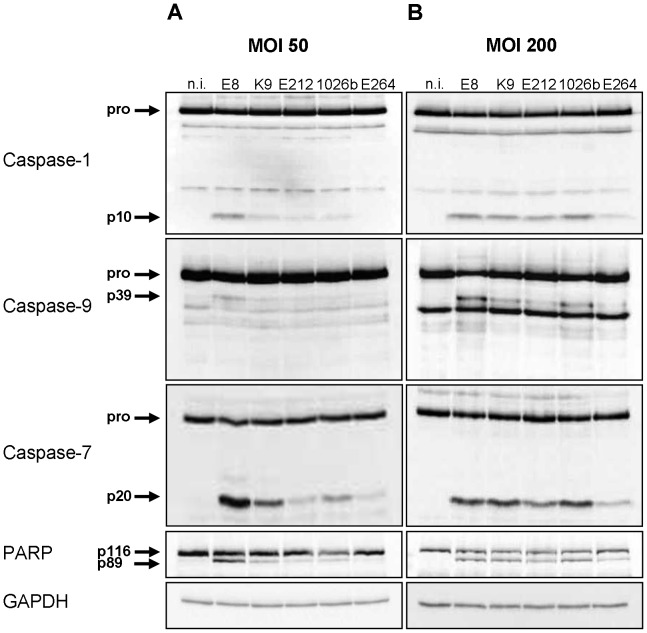
*Burkholderia*-mediated activation of caspase-1 in macrophages is strain- dependent. Cleavage of caspases-1, -9, -7, and PARP was detected by immunoblot in cell lysates of C57BL/6 wild-type BMM infected with *B. pseudomallei* strains E8, K96243 (K9), E212, 1026b, and *B. thailandensis* strain E264 at MOI of (**A**) 50∶1 and (**B**) 200∶1 at 1.5 hours post infection. One experiment of at least three performed is shown. non-infected (n.i.).

To examine caspase-1 activation and subsequent signalling pathways by environmental or clinical isolates of *Burkholderia*, wild-type bone marrow-derived macrophages (BMM) were infected with *B. pseudomallei* strains E8, E212, K96243 and 1026b as well as *B. thailandensis* strain E264 at multiplicity of infection (MOI) of 50 and 200, respectively. 1.5 hours after infection cells were harvested and lysates were analysed by immunoblotting with caspase-1, -9, -7, poly (ADP-ribose) polymerase (PARP), and glycerinaldehyde 3-phosphate dehydrogenase (GAPDH) antibodies. As shown in Figure 1A, infection of macrophages with MOI 50 resulted in a strong cleavage signal of caspases-1, -7 and PARP and a moderate activation of caspase-9 by *B. pseudomallei* strain E8, whereas processing was much less in response to *B. pseudomallei* strains K96243, 1026b, and E212. In contrast, infection of macrophages with *B. thailandensis* strain E264 just led to a weak cleavage signal of caspase-7 and PARP. However, infection of macrophages with MOI 200 resulted in a strong activation of caspases-1, -7 and cleavage of PARP and a minor activation of caspase-9 by all used *B. pseudomallei* strains compared to *B. thailandensis* strain E264 (Figure 1B). Thus, both *B. pseudomallei* strains and *B. thailandensis* strain E264 can activate caspase-1 signalling pathway in macrophages, but its activation seems to be strain-dependent.

### Caspase-1 restricts replication of both *B. pseudomallei* and *B. thailandensis* in macrophages

**Figure 2:**
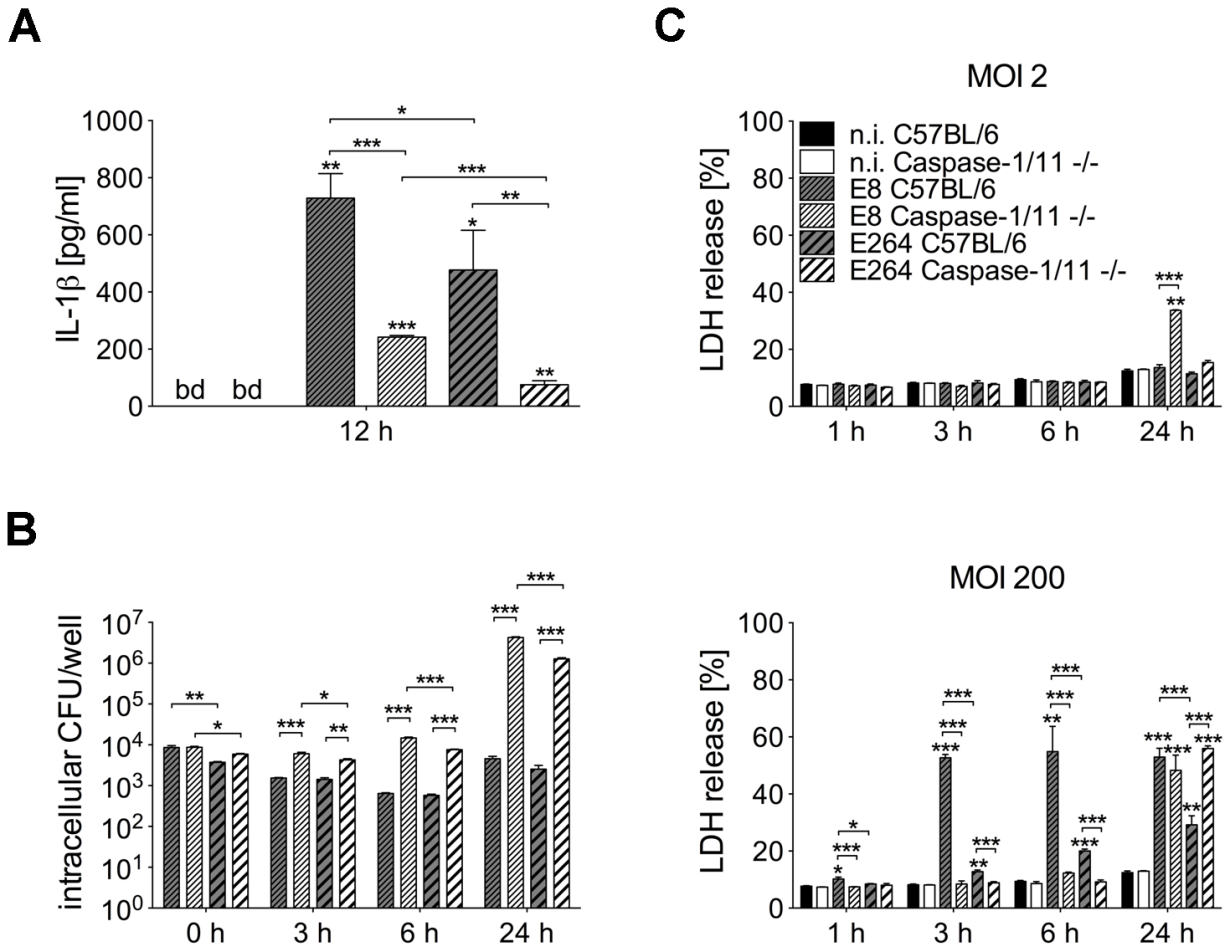
Course of cell death differs in caspase-1/11-deficient and wild-type macrophages infected with *Burkholderia*. (**A**) Secretion of IL-1β was determined in cell culture supernatants of BMM from caspase-1/11-deficient and C57BL/6 wild-type mice 12 hours after infection with *B. pseudomallei* E8 or *B. thailandensis* E264 (MOI 50∶1). (**B**) Invasion and intracellular bacterial growth of *B. pseudomallei* E8 and *B. thailandensis* E264 was examined in respective BMM infected at MOI of 2∶1 at the indicated time-points. (**C**) Induction of cytotoxicity was measured as lactate dehydrogenase (LDH) release in supernatants of *Burkholderia* infected caspase-1/11-deficient and wild-type BMM (MOI 2∶1 or 200∶1). (A) Data are presented as mean with standard error of the mean (SEM) of four independent experiments (n = 4). (B, C) Data are presented as mean with SEM of triplicate determinations. One representative experiment out of three independent experiments is shown. Statistical analyses were performed using one-way ANOVA (*p<0.05; **p<0.01; ***p<0.001 compared to non-infected macrophages or as indicated). below detection (bd), non-infected (n.i.).

To investigate whether differences in caspase-1 activation by *B. pseudomallei* strain E8 and *B. thailandensis* strain E264 might cause different IL-1β release, we determined the amount of the cytokine in cell culture supernatants of infected (MOI 50) and uninfected macrophages of caspase-1/11-deficient and wild-type mice at 12 hours post infection (Figure 2A). In macrophages of both mouse strains IL-1β concentrations were significantly higher in response to *B. pseudomallei* E8 compared to *B. thailandensis* E264 infection. As expected, secretion of IL-1β was strongly reduced but not completely abolished in caspase-1/11-deficient compared to wild-type macrophages. Hence, caspase-1/11 contributes to IL-1β secretion by macrophages, and the increased degree of caspase-1 activation by *B. pseudomallei* E8 might be responsible for higher IL-1β release compared to *B. thailandensis* E264.

We further compared invasion and intracellular survival of both *Burkholderia* strains in macrophages from caspase-1/11 knockout and control mice at low MOI (MOI 2) using kanamycin protection assay. Consistent with our previous study [29] the uptake of *B. pseudomallei* E8 was comparable in macrophages of both mouse strains and lack of caspase-1/11 was associated with an increase in bacterial burden already 3 hours after infection and even more pronounced 6 hours after infection. Remarkably, after 24 hours 1000-fold more bacteria were found in *B. pseudomallei*-infected caspase-1/11-deficient compared to wild-type macrophages (Figure 2B). *B. thailandensis* E264 showed a slightly lower invasion but a similar intracellular replication in caspase-1/11 knockout and control macrophages compared to *B. pseudomallei* E8 (Figure 2B). Therefore, caspase-1/11 may have an important role in controlling intracellular replication of both *B. pseudomallei* E8 and *B. thailandensis* E264 in macrophages.

### Course of cell death differs in caspase-1/11-deficient and wild-type macrophages after *Burkholderia* infection

To examine the induction of cell death in caspase-1/11 KO and wild-type macrophages by both *Burkholderia* species, cytosolic lactate dehydrogenase (LDH) activity was measured in cell culture supernatants of *B. pseudomallei* E8 or *B. thailandensis* E264 infected and uninfected macrophages at the indicated time points (Figure 2C). At low MOI (MOI 2) no differences in LDH release within 6 hours were detected in macrophages of both mouse strains infected with either *B. pseudomallei* E8 or *B. thailandensis* E264. In contrast, 24 hours after infection the cell damage was significantly more enhanced in caspase-1/11 KO macrophages infected with *B. pseudomallei* E8 compared to *B. thailandensis* E264. By using a high MOI (MOI 200), we found much more evident cell damage in wild-type than in caspase-1/11 KO macrophages within the early stages of infection, which is in agreement with results from our previous study [29]. Additionally, LDH release from macrophages infected with *B. pseudomallei* E8 was significantly higher compared to macrophages infected with *B. thailandensis* E264. However, 24 hours after infection, caspase-1/11 KO macrophages exhibited similar or even more LDH release in response to *B. pseudomallei* E8 and *B. thailandensis* E264, respectively. Our data indicate that infection with *B. pseudomallei* E8 and *B. thailandensis* E264 leads to an early, dose-dependent cell death in wild-type macrophages but to a delayed cell death in caspase-1/11 KO macrophages.

### Absence of caspase-1/11 results in cell destruction by activation of the apoptotic pathway in response to *Burkholderia* infection

**Figure 3:**
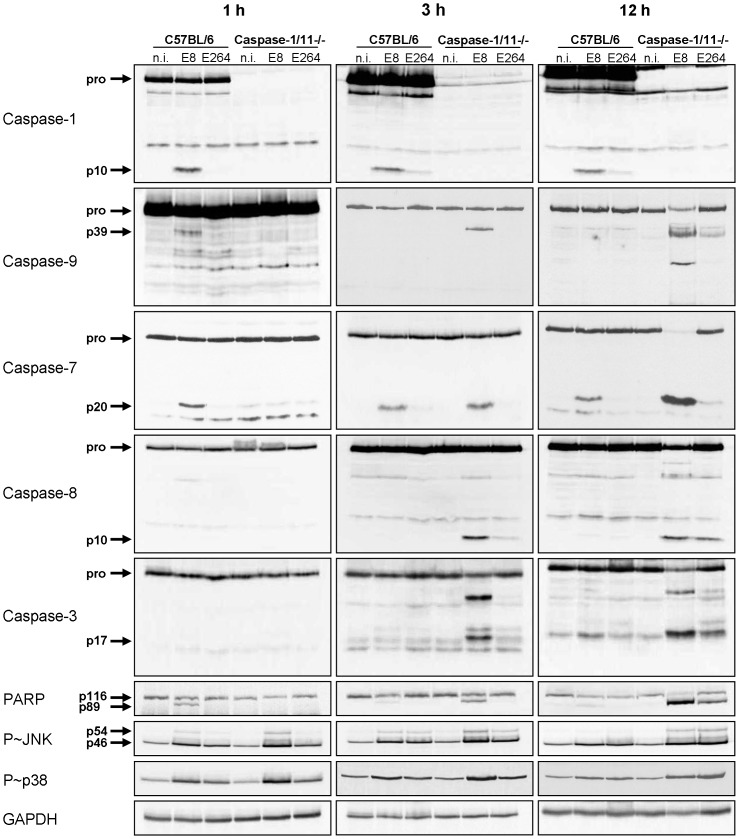
Caspase-1/11-deficient macrophages show activation of classical apoptotic pathways in response to *Burkholderia* infection. Processing of caspases-1, -9, -7, -8, -3, and PARP and phosphorylation of JNK and p38 MAPK was detected by immunoblot in cell lysates of caspase-1/11-deficient and wild-type BMM infected with *B. pseudomallei* E8 and *B. thailandensis* E264 at MOI of 50∶1 at one, three, and twelve hours after infection. One experiment of at least three performed is shown. non-infected (n.i.).

We previously reported that caspase-1/11 KO macrophages show activation of caspase-3 within 4 hours after *B. pseudomallei* E8 infection, whereas no significant caspase-3 activity could be measured in the wild-type [29]. To further analyse the underlying mechanisms of the different courses of cell death in caspase-1/11 KO and wild-type macrophages, we determined the activation of inflammatory caspase-1, classical apoptotic caspases as well as the phosphorylation of apoptosis-related kinases at different time points after infection with *B. pseudomallei* E8 or *B. thailandensis* E264 by immunoblotting. One hour post infection cleaved caspases-1, -9, -7 and PARP were found in wild-type macrophages infected with *B. pseudomallei* E8 but not with *B. thailandensis* E264 (Figure 3). However, processing could not be detected in caspase-1/11-deficient macrophages at this time point. Furthermore, there was no activation of caspase-8 and -3 neither in the wild-type nor in the caspase-1/11 knockout. Three and twelve hours after infection with *B. pseudomallei* E8 led to a stronger caspase-1 and -7 activation compared to *B. thailandensis* E264 in the wild-type as well as to a strong cleavage signal of caspase-7 in the caspase-1/11 knockout indicating that activation of caspase-7 is independent of caspase-1/11 at later stages of infection. Furthermore, infection of macrophages lacking caspase-1/11 resulted in an increased activation of apoptotic caspases-9, -8 and -3 in response to *B. pseudomallei* E8 and *B. thailandensis* E264. In line with these observations, infection of macrophages with *B. pseudomallei* E8 caused a higher phosphorylation rate of c-Jun-N-terminal kinase (JNK) and p38 mitogen-activated protein kinases (p38 MAPK), which are both involved in apoptotic signalling pathways, compared to *B. thailandensis* E264, whereas phosphorylation signals were stronger in caspase-1/11-deficient compared to wild-type macrophages. Our results indicate that at least two different types of cell death are induced in macrophages; an early caspase-1/11-dependent pyroptotic cell death in the wild-type and a delayed apoptotic cell death in the caspase-1/11 knockout, which is dependent on activation of apoptotic initiator and effector caspases.

### NLRC4-dependent caspase-1 activation is required for processing of caspases-9, -7 and PARP at early stages of infection

**Figure 4:**
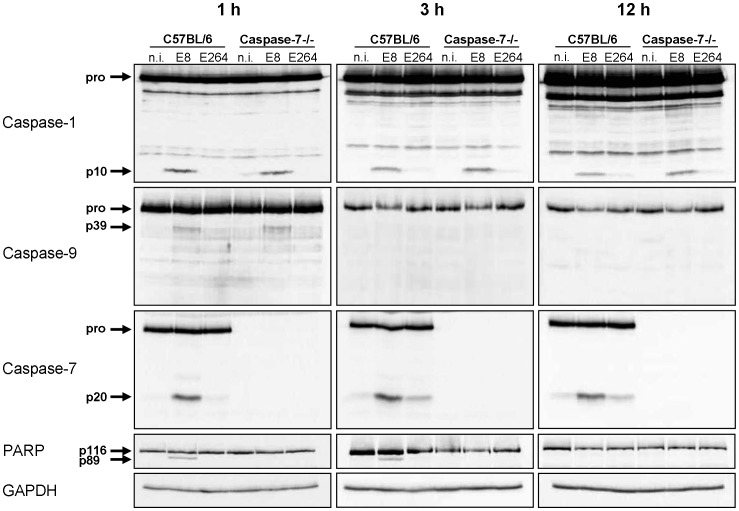
Early activation of caspase-7 in *Burkholderia* infected macrophages is dependent on caspase-1 and -9 and is essential for cleavage of PARP. Processing of caspases-1, -9, -7, and PARP was detected by immunoblot in cell lysates of caspase-7- deficient and C57BL/6 wild-type BMM infected with *B. pseudomallei* E8 and *B. thailandensis* E264 at MOI of 50∶1 at one, three, and twelve hours after infection. One experiment of at least three performed is shown. non-infected (n.i.).

**Figure 5:**
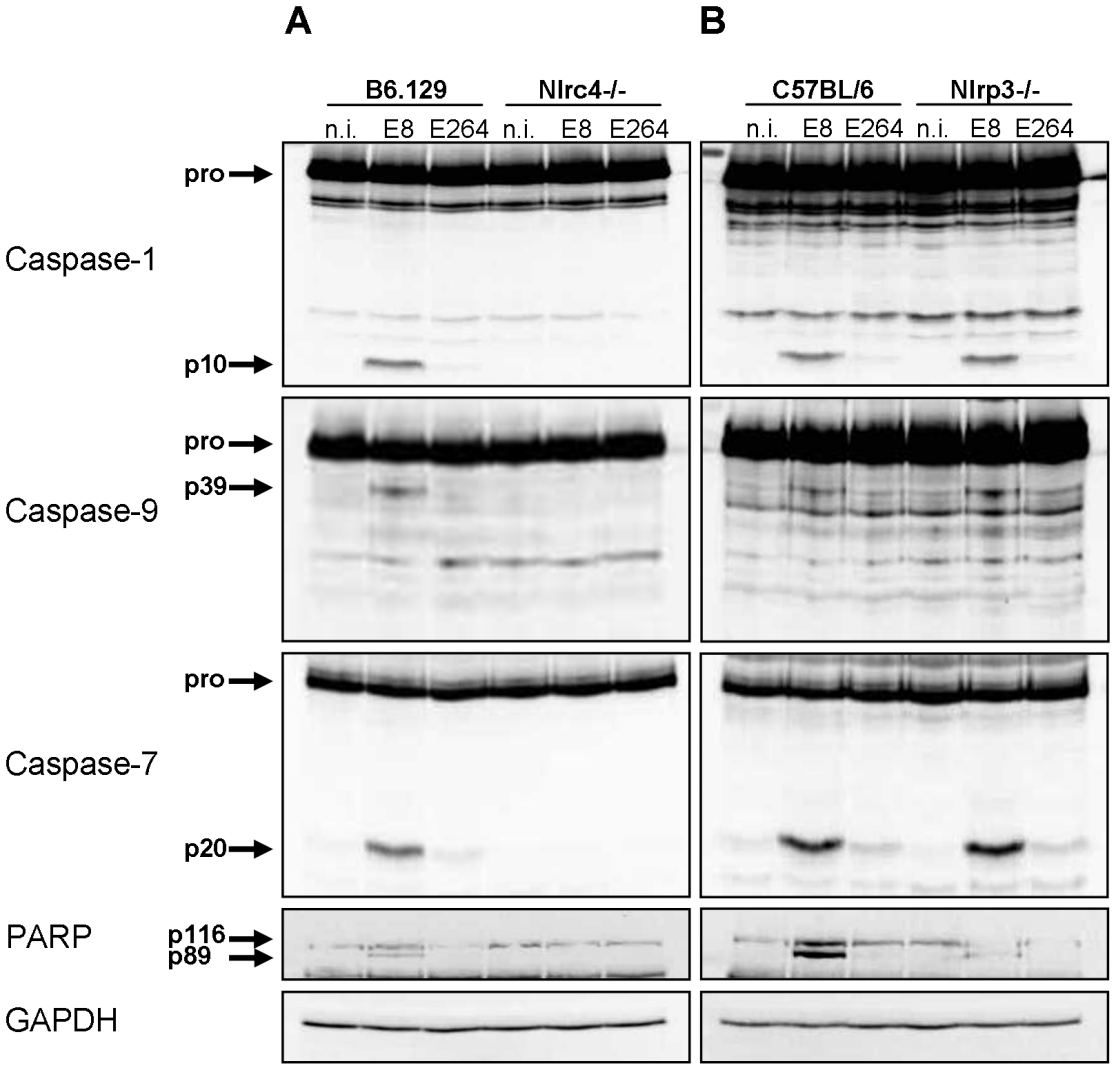
NLRC4, but not NLRP3 is required for early activation of caspase-1 in macrophages in response to *Burkholderia*. Processing of caspases-1, -9, -7 and PARP was detected by immunoblot in cell lysates of (**A**) NLRC4-deficient, (**B**) NLRP3-deficient and respective wild-type BMM infected with *B. pseudomallei* E8 and *B. thailandensis* E264 at MOI of 50∶1 at 1.5 hours post infection. One experiment of two performed is shown. non-infected (n.i.).

Since infection of wild-type macrophages caused a caspase-1-dependent activation of caspase-9 and -7 and cleavage of PARP, we investigated the order of processing downstream of caspase-1. Therefore, caspase-7 KO and wild-type macrophages were infected with *B. pseudomallei* E8 or *B. thailandensis* E264 and analysed for activation of caspases-1, -9, -7 and cleavage of PARP during different stages of infection. Whereas caspase-9 and -7 could not be activated in the caspase-1/11 knockout at one hour post infection (Figure 3), absence of caspase-7 did neither influence caspase-1 nor -9 processing (Figure 4). However, proteolytic inactivation of PARP was abolished in macrophages lacking caspase-7 at one and three hours after infection with *B. pseudomallei* E8. Thus, our data suggest that caspase-7 might act downstream of caspase-1/-9 but upstream of PARP. Pharmacological inhibition of caspase-9 in wild-type macrophages by treatment with Ac-LEHD-CHO did not prevent caspase-1 activation but failed to cleave caspase-7 and PARP after *B. pseudomallei* E8 infection (Figure S1) indicating that caspase-9 is activated downstream of caspase-1 but upstream of caspase-7/PARP. To analyse which NOD-like receptor is responsible for caspase-1 processing and signalling in the early phase, macrophages from wild-type mice or mice deficient in the inflammasome components NLRC4 and NLRP3 were infected with either *B. pseudomallei* E8 or *B. thailandensis* E264. Although activated caspase-1 was shown to be present in both NLRC4 and NLRP3 knockout macrophages eight hours after infection [12], we could not detect any processing of caspases-1, -9, -7 or PARP in macrophages lacking NLRC4 at 1.5 hours post infection, whereas NLRP3-deficient macrophages were still able to cleave caspases and PARP (Figure 5). But 12 hours post infection NLRC4-deficient and wild-type macrophages displayed similar caspase activation and PARP cleavage (Figure S2). Taken together we identified an activation pathway involving caspases-1, -9, -7 and PARP downstream of the host receptor NLRC4 in the early phase of infection with *B. pseudomallei* E8.

### *B. pseudomallei* FliC is involved in caspase-1 activation in macrophages but does not seem to be essential

**Figure 6:**
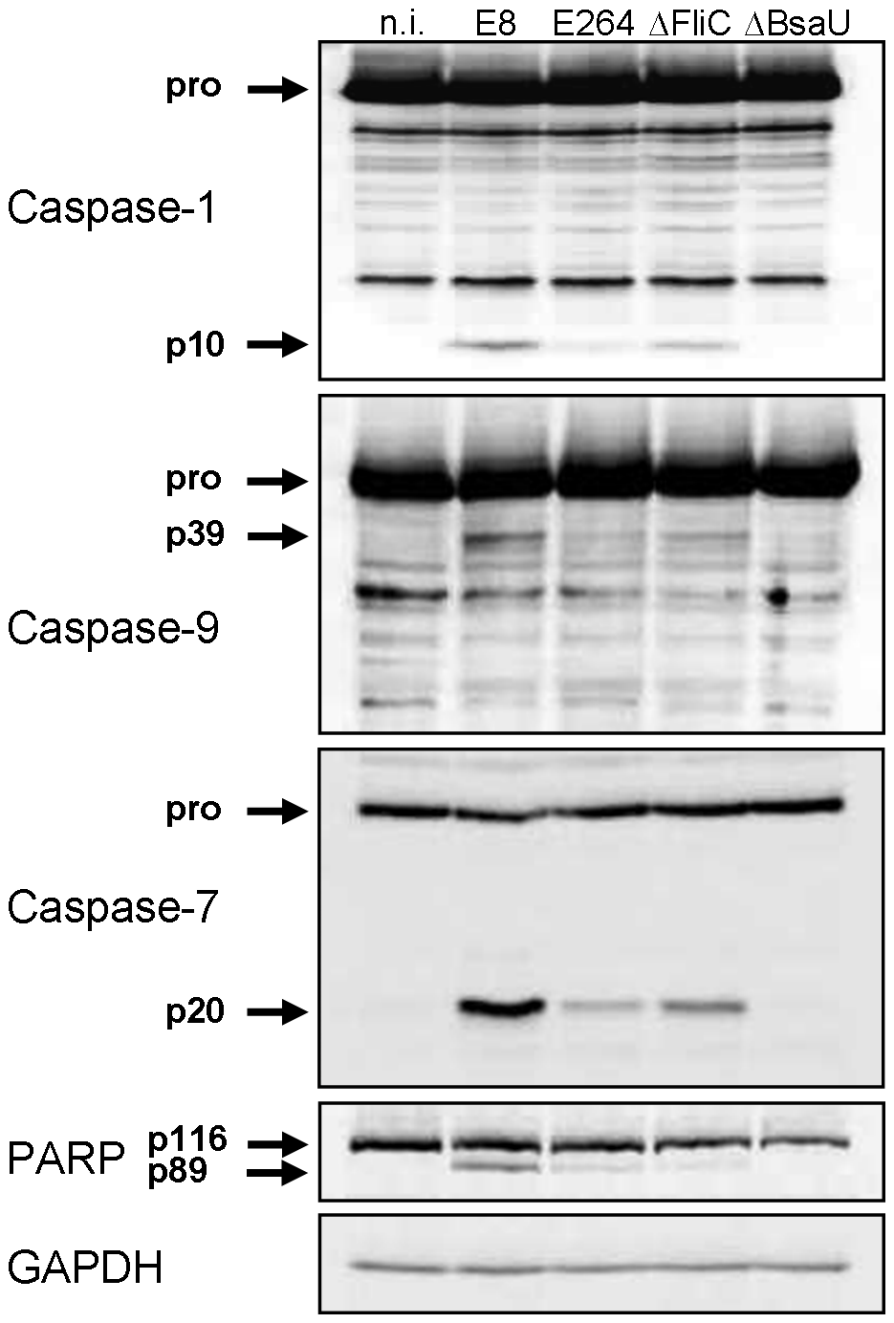
*B. pseudomallei* FliC contributes to caspase-1 activation in macrophages. Activation pattern of caspases-1, -9, -7 and PARP was detected by immunoblot in cell lysates of C57BL/6 BMM infected with *B. pseudomallei* E8, *B. thailandensis* E264 or *B. pseudomallei* E8 mutants FliC (ΔFliC) and BsaU (ΔBsaU) at MOI of 50∶1 at 1.5 hours post infection. One experiment of at least three performed is shown. non-infected (n.i.).

Several reports have shown that flagellin from many bacteria such as *S. typhimurium* or *L. pneumophila* acts as a potent inducer of the NLRC4 inflammasome [4], [5], [14]. In contrast, flagellin from *S. flexneri* or *B. thailandensis* did not stimulate NLRC4-dependent caspase-1 processing [13]. To determine the role of *B. pseudomallei* flagellin in caspase-1 activation, wild-type macrophages were infected with *B. pseudomallei* E8 or a flagellin-deficient *B. pseudomallei* E8 transposon mutant (ΔFliC). Figure 6 illustrates, that *B. pseudomallei* E8-induced caspase-1 activation at 1.5 hours post infection was only partially dependent on cytosolic flagellin as flagellin-deficient bacteria could still trigger caspase-1 cleavage although to a lesser extent compared to wild-type bacteria. As observed with caspase-1, also caspases-9, -7 and PARP showed an increased processing with flagellated *B. pseudomallei* E8 in comparison to flagellin-deficient bacteria. To further demonstrate that cytosolic presence of the pathogen is essential for caspase-1 activation, we included a *B. pseudomallei* E8 T3SS3 mutant that harbours a defect in the bsaU gene (BPSS1539). We previously found a transposon mutant of BsaU to be trapped within lysosomal-associated membrane protein-1 (LAMP-1) positive lysosomes after *B. pseudomallei* E8 infection and with a delayed escape into the cytosol [31], [32]. In the present study infection of macrophages with a deletion mutant of the putative needle length control protein BsaU (ΔBsaU) did not result in any processing of caspases or PARP in the early phase (Figure 6). Hence, our data indicate that in contrast to *B. thailandensis* E264, flagellin from *B. pseudomallei* E8 contributes to caspase-1 activation and a functional T3SS3 and/or cytosolic presence of the pathogen seems to be required.

### Transfection of *Burkholderia* BopE leads to caspase-1 activation in HEK293 cells

**Figure 7:**
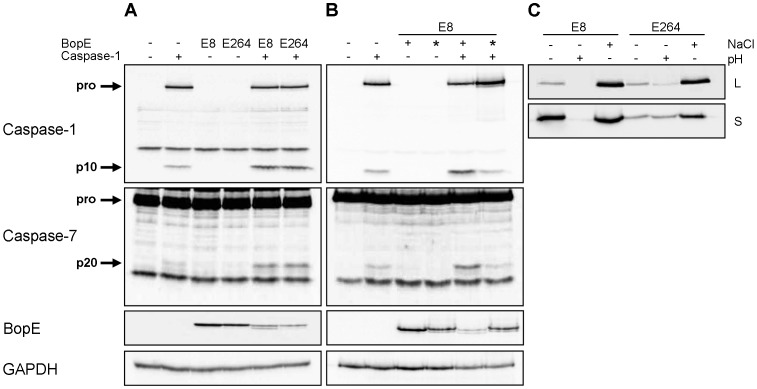
*Burkholderia* BopE is a mediator of caspase-1 activation in HEK293 cells. Processing of caspases-1, -7 and PARP and expression of BopE was detected by immunoblot in cell lysates of HEK293 cells transfected with (**A**) caspase-1-Flag, *B. pseudomallei* E8 BopE-Myc or *B. thailandensis* E264 BopE-Myc and (**B**) caspase-1-Flag, *B. pseudomallei* E8 BopE-Myc (+) or *B. pseudomallei* E8 BopE-R207E/N216P-Myc (*) as indicated. (**C**) BopE secretion in supernatants (S) and expression in cell lysates (L) were examined after growth of *B. pseudomallei* E8 and *B. thailandensis* E264 for 6 hours in LB broth (pH 7, 86 mM NaCl), LB broth at pH 4.5 (+) or at 320 mM NaCl (+). (A–C) One experiment of at least three performed is shown.

The *Burkholderia* T3SS3 effector protein BopE was found to share significant homology with the *Salmonella* T3SS effectors SopE and SopE2 [33], [34] activating caspase-1 in different cell types [17], [35] by the guanine nucleotide exchange factor (GEF) activity of SopE [36]. To determine whether *Burkholderia* BopE that was shown to have a GEF activity as well [33] alone is sufficient to activate caspase-1, HEK293 cells, which do not express full caspase-1, were cotransfected with expression plasmids for caspase-1 (caspase-1-Flag) and for BopE (bopE-Myc) of either *B. pseudomallei* E8 or *B. thailandensis* E264 and analysed for caspase processing. Transient cotransfection of caspase-1-Flag and *Burkholderia* bopE-Myc resulted in stronger caspase-1 and -7 activation compared to caspase-1-Flag and pcDNA-Myc, whereas transfection of the respective bopE-Myc alone did not cause any caspase activation (Figure 7A). Mutation of the GEF activity domain of *B. pseudomallei* E8 bopE-Myc (bopE-R207E/N216P) according to Upadhyay et al. [37] led to a decrease in caspase-1 and -7 activation to the basal level (Figure 7B) indicating that the GEF activity of *B. pseudomallei* BopE contributes to caspase-1 activation in HEK293 cells. To analyse whether differences in caspase-1 activation, IL-1β release and pyroptosis induction in murine macrophages by *B. pseudomallei* E8 and *B. thailandensis* E264 might be mediated by distinct BopE secretion of both *Burkholderia* species, we determined expression and secretion of BopE of both bacterial strains cultured in LB broth (pH 7; 86 mM NaCl), under acidic growth conditions (pH 4.5) and salt stress (320 mM NaCl). Western blot analysis indicated that BopE of *B. thailandensis* E264 was less secreted than BopE of *B. pseudomallei* E8 cultured in standard as well as NaCl supplemented LB broth (Figure 7C), which might account for the distinct capacity to activate caspase-1 in macrophages.

### *B. pseudomallei* BopE is dispensable for caspase-1 activation in macrophages

**Figure 8:**
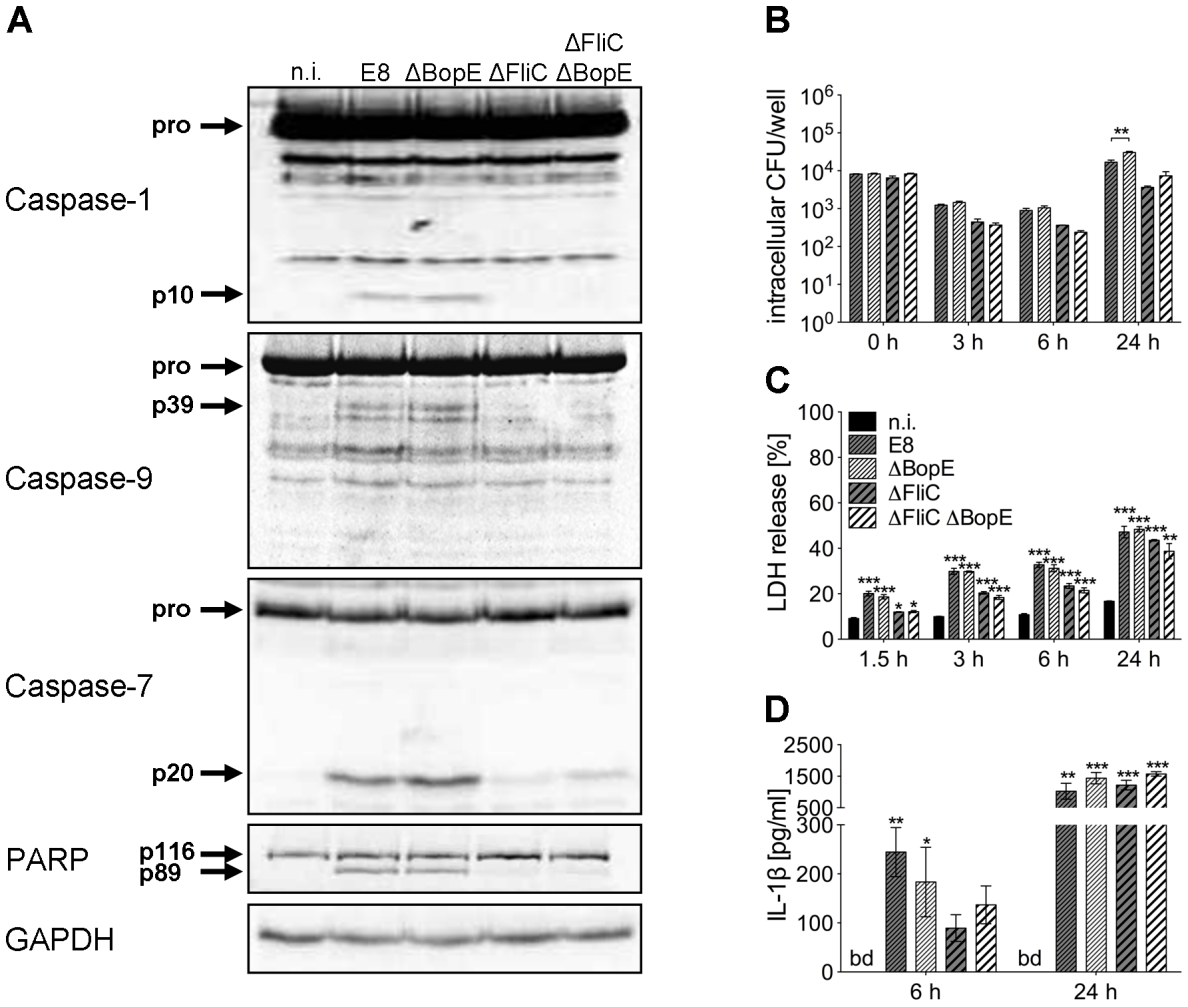
Deletion of *B. pseudomallei* BopE does not influence caspase-1 activation in macrophages. (**A**) Processing of caspases-1, -9, -7 and PARP was detected by immunoblot in cell lysates of C57BL/6 BMM infected with *B. pseudomallei* E8 wild-type or mutants ΔBopE, ΔFliC and ΔFliCΔBopE at MOI of 50∶1 at 1.5 hours post infection. One experiment of at least three performed is shown. non-infected (n.i.). (**B**) Invasion and intracellular replication of respective *B. pseudomallei* strains was examined in BMM infected at MOI of 2∶1 at the indicated time points. (**C**) Induction of pyroptosis was measured as lactate dehydrogenase (LDH) release in cell culture supernatants of *B. pseudomallei* infected BMM (MOI 200∶1). (**D**) Secretion of mature IL-1β was measured in supernatants of *B. pseudomallei* infected BMM (MOI 50∶1) at 6 and 24 hours after infection. (B, C) Data are presented as mean with standard error of the mean (SEM) of triplicate determinations. One representative experiment out of three independent experiments is shown. (D) Data are presented as mean with SEM of four independent experiments (n = 4). Statistical analyses were performed using one-way ANOVA (*p<0.05; **p<0.01; ***p<0.001 compared to non-infected macrophages or as indicated). below detection (bd), non-infected (n.i.).

To further characterize the role of BopE in murine macrophages, we generated two mutants of *B. pseudomallei* E8, lacking either BopE (ΔBopE) or flagellin FliC and BopE (ΔFliCΔBopE) by targeted mutagenesis. Figure 8A shows that *B. pseudomallei* ΔBopE was still able to activate caspases-1, -9, -7 and to cleave PARP as strong as wild-type bacteria. As expected macrophages infected with ΔFliC but also ΔFliCΔBopE caused less caspase-1 activation compared to wild-type bacteria. Furthermore, we examined the role of BopE in invasion and intracellular replication by infecting macrophages with wild-type bacteria and the mutant strains. Within 6 hours after infection ΔBopE showed a similar intracellular replication compared to the wild-type but after 24 hours the ability to multiply inside macrophages was slightly but significant increased after infection with the BopE mutant compared to the wild-type (Figure 8B). Next we assessed whether BopE might be involved in the induction of pyroptosis by measuring the release of LDH at various time points after infection. However, there was no significant difference between ΔBopE and wild-type bacteria in the LDH release at any time (Figure 8C). Finally, we quantified secretion of IL-1β by macrophages infected with wild-type bacteria or mutant strains. But we did not detect significant difference in IL-1β secretion, although mutation of BopE resulted in slightly higher IL-1β release at 24 hours after infection (Figure 8D). These results indicate that the *B. pseudomallei* BopE caspase-1 processing capacity does not seem to play a major role during macrophage infection.

### *B. pseudomallei* BsaK contributes to caspase-1 activation, pyroptosis and IL-1β secretion in macrophages

**Figure 9:**
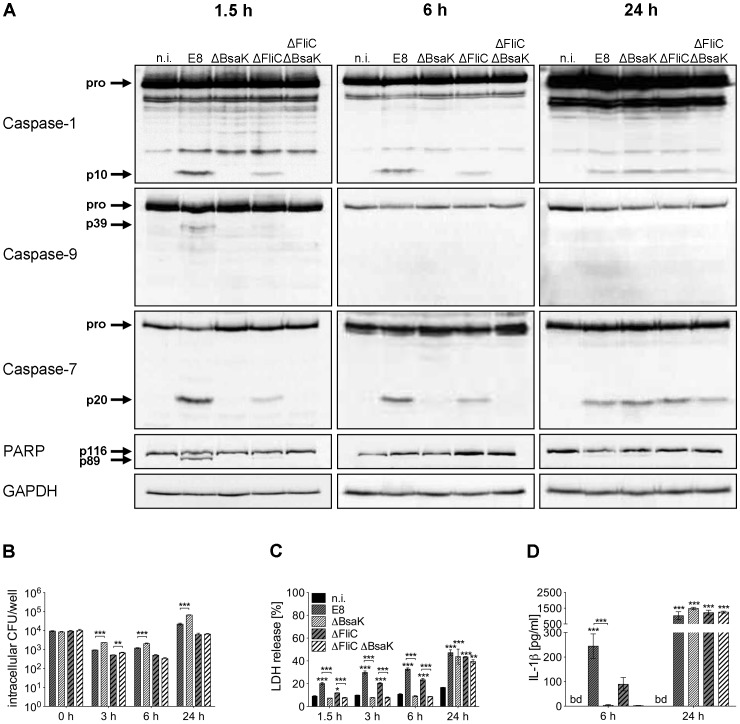
*B. pseudomallei* BsaK is involved in caspase-1-dependent pyroptosis and IL-1β production in macrophages in the early phase of infection. (**A**) Processing of caspases-1, -9, -7 and PARP was detected by immunoblot in cell lysates of C57BL/6 BMM infected with *B. pseudomallei* E8 wild-type or mutants ΔBsaK, ΔFliC and ΔFliCΔBsaK at MOI of 50∶1 at 1.5, 6 and 24 hours post infection. One experiment of at least three performed is shown. non-infected (n.i.). (**B**) Invasion and intracellular replication of respective *B. pseudomallei* strains was examined in BMM infected at MOI of 2∶1 at the indicated time points. (**C**) Induction of pyroptosis was measured as lactate dehydrogenase (LDH) release in cell culture supernatants of *B. pseudomallei* infected BMM (MOI 200∶1). (**D**) Secretion of mature IL-1β was measured in supernatants of *B. pseudomallei* infected BMM (MOI 50∶1) at 6 and 24 hours after infection. (B, C) Data are presented as mean with standard error of the mean (SEM) of triplicate determinations. One representative experiment out of three independent experiments is shown. (D) Data are presented as mean with SEM of four independent experiments (n = 4). Statistical analyses were performed using one-way ANOVA (*p<0.05; **p<0.01; ***p<0.001 compared to non-infected macrophages or as indicated). below detection (bd), non-infected (n.i.).

A recent study identified the T3SS3 inner rod protein BsaK of *B. pseudomallei* as a stimulator of the NLRC4 inflammasome [11]. To further characterize the role of BsaK in caspase-1 activation, we generated two mutants of *B. pseudomallei* E8, lacking either BsaK (ΔBsaK) or flagellin FliC and BsaK (ΔFliCΔBsaK) followed by infection of macrophages. In contrast to wild-type bacteria, both ΔBsaK and ΔFliCΔBsaK were not able to activate caspases-1, -9, -7 and to cleave PARP 1.5 hours after infection (Figure 9A), indicating that *B. pseudomallei* BsaK is required for early caspase-1 activation. At 6 hours after infection we also found much less caspase-1 and -7 cleavage signals in ΔBsaK or ΔFliCΔBsaK infected BMM compared to wild-type infected BMM. However, after 24 hours there was no difference in activation of both caspases between these strains anymore (Figure 9A) suggesting that caspase-1 activation in the late phase of infection is BsaK-independent. Moreover, we investigated the role of BsaK in invasion and intracellular replication by infecting macrophages with wild-type bacteria and the respective mutant strains. There was no difference in invasion of macrophages between mutant strains and the wild-type. However, 3, 6 and 24 hours after infection ΔBsaK showed higher intracellular counts compared to the wild-type (Figure 9B). ΔFliCΔBsaK exhibited increased intracellular growth only at 3 hours post infection in comparison to ΔFliC. At early time points (≤6 h post infection) we found ΔBsaK and ΔFliCΔBsaK to cause significantly reduced LDH release in macrophages compared to the wild-type strain. But 24 hours after infection ΔBsaK, ΔFliCΔBsaK and the wild-type strain showed comparable release of LDH (Figure 9C). Therefore, BsaK seems to contribute to pyroptotic cell death in the early phase of *B. pseudomallei* infection. In addition, absent caspase-1 processing by ΔBsaK and ΔFliCΔBsaK was attendend by significantly reduced secretion of IL-1β six hours after infection, whereas a similar IL-1β production could be detected 24 hours after infection (Figure 9D). As a *B. pseudomallei* mutant lacking FliC still activated caspase-1 in wild-type macrophages but a BsaK mutant did not, our data support a major importance of a functional *Burkholderia* T3SS3 for caspase-1 activation, pyroptosis induction and IL-1β secretion.

### *B. pseudomallei* BsaK mutant is attenuated in BALB/c mice

**Figure 10:**
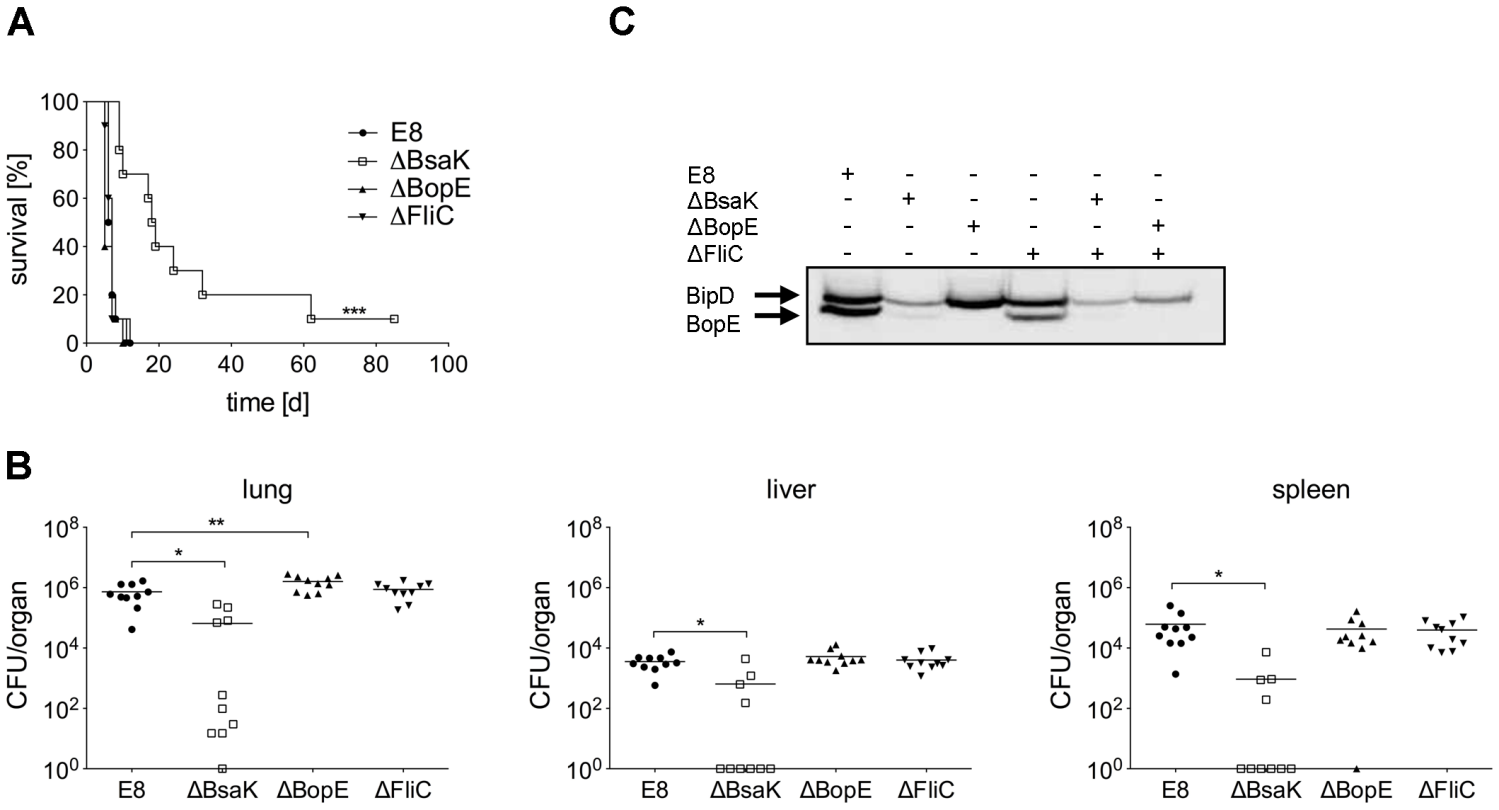
*B. pseudomallei* BsaK mutant is attenuated in a pulmonary mouse model. (**A**) BALB/c mice were intranasally infected with *B. pseudomallei* E8 wild-type, or mutants ΔBsaK, ΔBopE, and ΔFliC at 40 CFU and survival was monitored (log rank Kaplan-Meier test, ***p<0.001 compared to *B. pseudomallei* E8 wild-type). Pooled data from two independent experiments are shown (n = 10). (**B**) Mice were sacrificed 48 hours after infection and the bacterial load (CFU) in lung, liver and spleen was determined. Pooled data from two independent experiments are presented as mean (n = 10). Statistical analyses were performed using one-way ANOVA (*p<0.05; **p<0.01). (**C**) Secretion of BipD and BopE was examined after growth of *B. pseudomallei* E8 wild-type, or mutants ΔBsaK, ΔBopE, ΔFliC, ΔFliCΔBsaK and ΔFliCΔBopE for 6 hours in LB broth. One representative experiment out of two independent experiments is shown.

**Figure 11:**
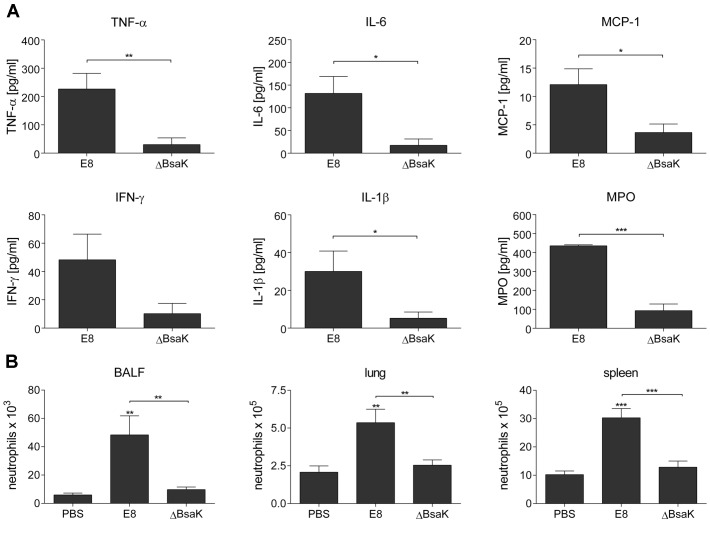
*B. pseudomallei* ΔBsaK infected mice show reduced cytokine levels and neutrophil influx. BALB/c mice were intranasally infected with *B. pseudomallei* E8 wild-type and ΔBsaK at 40 CFU. (**A**) Cytokine (TNF-α, IL-6, MCP-1, IFN-γ, IL-1β) and myeloperoxidase (MPO) levels were measured in BALF obtained 48 hours post infection. (**B**) Flow cytometric analyses for neutrophils in BALF, lung and spleen was performed 48 hours after challenge with *B. pseudomallei* E8 wild-type, ΔBsaK or PBS. (A, B) Pooled data from two independent experiments are presented as mean with standard error of the mean (n = 10). Statistical analyses were performed using (A) a Student's *t* test or (B) one-way ANOVA (*p<0.05; **p<0.01; ***p<0.001 compared to PBS infected mice or as indicated).

To examine whether mutation of *B. pseudomallei* FliC, BopE or BsaK is associated with *in vivo* virulence, we infected BALB/c mice intranasally with approx. 40 CFU of either wild-type or mutant strain. Mice infected with wild-type (median survival: 6.5 d), ΔBopE (median survival: 5 d) or ΔFliC (median survival: 7 d) bacteria succumbed within the first days after infection whereas ΔBsaK infected mice survived significantly longer (median survival: 18.5 d) (Figure 10A). Increased survival of ΔBsaK infected mice was attended by significant lower bacterial burden in lung, liver and spleen 48 hours after infection compared to wild-type infected mice (Figure 10B). In contrast, there was no difference in bacterial load in spleen and liver after infection with both ΔFliC and ΔBopE, whereas ΔBopE showed higher bacterial numbers in the lung compared to the wild-type. To analyse whether differences in survival and bacterial burden might be mediated by distinct secretion of T3SS3 needle tip protein BipD or effector protein BopE, we determined release of both proteins by *B. pseudomallei* wild-type and mutant strains cultured in LB broth. Western blot analysis indicated that BipD and BopE were less produced by ΔBsaK and ΔFliCΔBsaK compared to wild-type bacteria or ΔFliC. In contrast, BipD expression was not different in ΔBopE and ΔFliCΔBopE (Figure 10C). To further characterize *in vivo* effects of the *B. pseudomallei* BsaK mutant, we examined cytokine secretion in both bronchoalveolar lavage fluids (BALF) and serum of ΔBsaK and wild-type infected mice 48 hours after infection. ΔBsaK infected mice secreted significantly lower levels of IL-1β as well as TNF-α, IL-6, MCP-1 and IFN-γ compared to the wild-type strain (Figures 11A, S3). In addition, lower concentrations of the neutrophil enzyme myeloperoxidase (MPO) were detected in BALF of ΔBsaK compared to wild-type infected mice (Figure 11A). Recruitment of neutrophils (Figure 11B) and dendritic cells (Figure S9) into BALF and lung as well as spleen was significantly decreased in mice infected with the BsaK mutant.

To confirm results with the *B. pseudomallei* BsaK mutant obtained in the respiratory mouse model, we infected BALB/c mice intravenously with approx. 250 CFU of either wild-type or BsaK mutant. We found lower bacterial numbers in lung, liver and spleen 48 hours after infection compared to wild-type infected mice (Figure S4). In addition, ΔBsaK infected mice secreted significantly lower levels of MCP-1, but in contrast to the pulmonary model higher levels of IFN-γ and IL-6 compared to the wild-type strain (Figure S4). Thus, our results indicate that BsaK mediates caspase-1 activation and downstream effector mechanisms in primary macrophages, but at the same time contributes to virulence in a respiratory and systemic model of murine melioidosis.

## Discussion

The flagellate bacterium *B. pseudomallei* is able to escape from the vacuole, to replicate in the cytosol of eukaryotic cells and to activate the host inflammasome. We previously described that inflammatory caspase-1 mediates resistance in murine melioidosis as caspase-1/11 knockout mice reveal increased mortality compared to control mice by intranasal challenge with *B. pseudomallei* E8. Furthermore, macrophages deficient for caspase-1/11 showed impaired bactericidal activity in comparison to wild-type macrophages [29]. In the present study we found that activation of caspase-1 occurs early in the infection process and is dependent on the *Burkholderia* strain. Besides caspase-1 we observed subsequent activation of caspase-9 and -7 in wild-type macrophages after infection with *B. pseudomallei* E8 (Figure 1). As pharmacological inhibition of caspase-9 compromised caspase-7 processing by *B. pseudomallei* (Figure S1), we suggest that caspase-7 is dependent on caspase-9. Previous studies have implicated the cleavage of caspase-7 by caspase-1 as a consequence of inflammatory stimulation or infection with *S. typhimurium* [20] or *L. pneumophila* [21], two gram-negative pathogens which naturally reside in the vacuole. Thus, we asked whether caspase-7 might act downstream of caspase-1 in response to cytosolic *B. pseudomallei* infection as well. Using caspase-1/11 and -7 knockout macrophages we found that at early time points *B. pseudomallei* mediated caspase-7 activation was abolished in absence of caspase-1/11 (Figure 3), whereas caspase-1 activation did not require caspase-7 (Figure 4). This result indicates that caspase-7 is downstream of the caspase-1/9 activation pathway. However, at later time points caspase-7 could be also activated independently of caspase-1 and -9 in response to *B. pseudomallei* (Figure 3). In accordance with these findings, recently published work showed that activation of caspase-7 during infection with gram-positive *L. monocytogenes* does not require caspase-1 [38].

So far little is known about the biological function of caspase-7 activation downstream of caspase-1. A previous study demonstrated that processing of caspase-7 leads to control of *L. pneumophila* intracellular growth due to its contribution to bacteria phagosome maturation in macrophages and rapid cell death during early stages of infection [21]. In contrast, we could not detect any significant difference in intracellular replication of *B. pseudomallei* between caspase-7-deficient and wild-type macrophages (Figure S5B). However, we measured slightly increased LDH release in the caspase-7 knockout compared to the wild-type (Figure S5C). Accordingly, caspase-1-independent caspase-7 activation was shown to be a protective host response to plasma membrane damage caused by pore-forming toxins during *L. monocytogenes* infection limiting cytotoxicity [38].

In addition to processing of caspases-1, -9 and -7 in wild-type macrophages, we also found cleavage of the DNA damage sensor PARP (Figure 1), which is a known feature of apoptosis. But a recent study revealed that caspase-1 and downstream effector caspase-7 are also responsible for cleavage of PARP upon inflammasome stimulation contributing to pyroptotic cell death [39]. As NLRC4 was shown to be essential for *S. typhimurium*-mediated caspase-1 activation and pyroptosis induction in macrophages [5], [40], *S. typhimurium*-induced PARP processing was abrogated in NLRC4-deficient macrophages [39]. In agreement with these observations, we found that macrophages deficient for caspases-1, -7 or -9 (Figures 3, 4, S1) and those lacking inflammasome receptor NLRC4 (Figure 5A) are highly resistant to cleavage of PARP compared to wild-type macrophages in response to *B. pseudomallei* infection. Thus, NLRC4-dependent activation of caspases-1, -9, and -7 is essential for *Burkholderia*-dependent PARP proteolytic inactivation during pyroptosis. On that score it was suggested that PARP processing might be a general strategy used by cells undergoing programmed cell death to conserve the cellular energy stores to permit precise execution of the cell death program [39]. Furthermore, a recent study revealed that inflammasome-activated caspase-7 can translocate to the nucleus and cleave PARP to enhance expression of a subset of NFκB target genes negatively regulated by PARP including GM-CSF, IL-6 and LIF [41]. However, in the present study we could not find any significant difference of IL-1β (Figure S5A), IL-6 or TNF-α expression (data not shown) in *B. pseudomallei* infected caspase-7-deficient compared to wild-type macrophages. Moreover caspase-7-deficient mice showed no significant differences in bacterial burden in organs or serum cytokines after intranasal challenge with *B. pseudomallei*, but exhibited a slightly increased mortality rate compared to wild-type mice (median survival: 13 d vs. 34.5 d) (Figure S6). Thus, the biological function of caspase-7 in *B. pseudomallei* infection remains elusive.

Previous work by Ceballos-Olvera et al. [12] revealed that *B. pseudomallei* can activate the host inflammasome by the NOD-like receptors NLRC4 and NLRP3. They demonstrated that both NLRC4 and NLRP3 knockout macrophages can cleave caspase-1 as wild-type macrophages eight hours after infection. In contrast, our study showed that infection of wild-type (Figure 1) and NLRP3 knockout macrophages (Figure 5B) but not NLRC4-deficient macrophages (Figure 5A) with *B. pseudomallei* E8 leads to activation of caspase-1 and downstream signalling pathways at 1.5 hours after infection. This suggests that caspase-1 processing is exclusively dependent on NLRC4 inflammasome in the early phase of *B. pseudomallei* infection. Pyroptosis functions to lyse compromised macrophages and dendritic cells that harbor bacteria capable of replicating in the intracellular niche [18]. In accordance, we found that NLRC4 and caspase-1 are important for induction of pyroptotic cell death (Figures S7A, 2C) regulating intracellular growth of *Burkholderia* (Figures S7A, 2B) early in the infection process. A recent published study showed that not only caspase-1 but also caspase-11 plays a critical role in limiting *Burkholderia* infection. It was observed that caspase-11 promotes pyroptosis without IL-1β secretion in response to cytosolic but not to vacuolar bacteria detected through a hypothetical non-canonical inflammasome [30]. In addition, several studies have reported that caspase-11 may cause caspase-1-independent cell death in response to a number of bacterial pathogens including *S. typhimurium* and *L. pneumophila* [42]–[46] in a flagellin-independent manner [44], [47]. However, although a role of caspase-11 in pyroptosis induction was recently shown, we were not able to detect any cleavage of caspase-11 in response to *B. pseudomallei* E8 or *B. thailandensis* E264 at 1.5 (Figure S8), 3 and 12 hours (data not shown) after infection of macrophages. Since NLRC4-induced pyroptosis did require neither ASC nor the cleavage of procaspase-1 [48], this might apply to caspase-11 as well.

The extent and the course of macrophage death seem to be strongly dependent on the infection dose. In line with our previous study [29], we observed no differences in LDH release within 6 hours after infection at low MOI between caspase-1/11-deficient and wild-type macrophages (Figure 2C), but a 10-fold higher bacterial burden in cells lacking caspase-1/11 (Figure 2B) indicating that caspase-1-dependent effector functions might contribute to the control of *B. pseudomallei* intracellular replication. Rapid NLRC4- and caspase-1-dependent cell death in wild-type macrophages at high MOI led to high LDH release within the first hours after infection compared to NLRC4 and caspase-1/11 knockout macrophages, which exhibited signs of cell death as recently as 24 hours (Figures S7A, 2C). Therefore, we suggest that at least two different types of cell death are induced in macrophages; an early NLRC4-caspase-1/11-dependent pyroptotic cell death in the wild-type and a delayed apoptotic cell death in the caspase-1/11 knockout. In agreement, we detected a strong activation of classical apoptotic initiator (caspases-9, -8) and effector (caspases-3, -7) caspases as well as phosphorylation of apoptosis-related kinases p38 MAPK and JNK in caspase-1/11-deficient macrophages after infection with *B. pseudomallei* (Figure 3) indicating that in the absence of caspase-1/11, alternate cell death pathways such as apoptosis might compensate for the lack of pyroptosis. Accordingly, caspase-1 knockout macrophages infected with *S. typhimurium* have been shown to die via variable cell death pathways including apoptosis [49], [50] or autophagy [51] that are kinetically much slower than pyroptosis. Subsequently, it was demonstrated that in absence of caspase-1, recognition of DNA released by the cytosolic pathogen *Francisella tularensis* subspecies *novicida* via the absent in melanoma 2 (AIM2) inflammasome can trigger ASC- mediated caspase-8 activation and apoptotic cell death to restrict intracellular replication *in vitro* [52]. A recently published work showed that AIM2 and NLRP3 inflammasomes can activate caspase-8 and caspase-1 leading to both apoptotic and pyroptotic cell death pathways via ASC by cytosolic DNA or bacterial pore-forming toxin nigericin, respectively. The balance between both forms of programmed cell death seems to be dependent on the amount of bacterial DNA, with lower DNA concentrations seen in apoptosis and higher concentrations seen in pyroptosis [53]. Taken together, these studies emphasize the interplay between inflammasome components and the apoptotic apparatus and illustrate the possible switch between pyroptotic and apoptotic pathways upon PRR engagement [52].

To date, a number of *B. pseudomallei* factors that play a role in virulence including the capsular polysaccharide [54], [55], the quorum-sensing system or the type three [34], [56] and type six [57], [58] secretion systems have been described but the factors contributing to inflammasome-mediated caspase-1 activation are poorly characterized. In the present study transfection of *Burkholderia* T3SS3 effector protein BopE, which is homologous to the *Salmonella* T3SS effectors SopE and SopE2 [33], [34] into HEK293 cells led to stronger caspase-1 activation compared to the control (Figure 7A). Because mutation of the guanine nucleotide exchange factor activity of *B. pseudomallei* BopE resulted in a decrease in caspase-1 activation to the basal level (Figure 7B), this points to a role of the BopE GEF activity in activation of caspase-1 in HEK293 cells. In line with these reports, SopE has been reported to function as highly efficient GEF activating Rho GTPases [36], [59] to cause host cell invasion, caspase-1 activation and IL-1β release *in vitro* [17]. As BopE of *B. thailandensis* E264 was shown during our study to be much less secreted than BopE of *B. pseudomallei* E8 (Figure 7C), the observed differences in activation of caspase-1 (Figure 1) and induction of pyroptosis in macrophages (Figure 2C) might be mediated by distinct BopE secretion of both *Burkholderia* species. Although *Salmonella* SopE was shown to contribute to caspase-1 activation in RAW264.7 macrophage cells [35], deletion of *B. pseudomallei* BopE did not impair caspase-1 activation and signalling in primary macrophages (Figure 8A). Furthermore, we could not find an essential role for BopE in *B. pseudomallei* invasion (Figure 8B), induction of pyroptosis (Figure 8C) or IL-1β release (Figure 8D) in macrophages, whereas a previous study reported that *B. pseudomallei* 10276 BopE is required for optimal invasion of non-phagocytic HeLa cells analysed six hours after infection [33]. Thus, we propose that BopE, although per se capable to activate caspase-1, is dispensable for caspase-1 processing in *B. pseudomallei* infected macrophages since other bacterial factors might substitute this function and might contribute to caspase-1 activation to a larger extent. Consistent with our *in vitro* studies we could not discover any role of BopE during intranasal infection (Figure 10). This result agrees with previous work indicating that a *B. pseudomallei* 576 BopE mutant showed hardly any virulence attenuation during intraperitoneal infection of BALB/c mice [60]. In contrast, *Salmonella* SopE was reported to cause mucosal inflammation *in vivo* thereby reducing bacterial burden [17].

Several reports revealed that cytosol-delivered flagellin of several bacteria is capable of activating caspase-1 in macrophages by NLRC4 [4], [5], [8], [35]. Flagellin of *S. typhimurium* and *P. aeruginosa* but not *B. thailandensis* was shown to interact with NAIP5 stimulating NLRC4-dependent caspase-1 activation, macrophage death and IL-1β production [13]. Sun and Gan demonstrated that induction of caspase-1 processing by *B. pseudomallei* required intact T3SS3 [28] but not flagellin [61]. We found that early processing of caspase-1 (Figures 6, 8A, 9A), induction of pyroptosis (Figures 8C, 9C) and secretion of IL-1β (Figures 8D, 9D) were reduced after infection of BMM with a *B. pseudomallei* flagellin FliC mutant. These results indicate that *B. pseudomallei* flagellin contributes to caspase-1 activation during infection. Although flagella are considered as a virulence determinant in many bacteria, their role in the pathogenesis of *B. pseudomallei* is discussed controversially. Previous reports suggest that flagella may be of significance for *B. pseudomallei* motility but are not important for invasion of *B. pseudomallei* into mouse macrophage cells or human lung epithelial cells during *in vitro* assays using centrifugation to mediate pathogen-host cell contact [62]–[64]. In accordance with these reports no different [62], [63] or only slightly reduced (Figures 8B, 9B; [64]) bacterial replication rates of flagellin-deficient compared to wild-type bacteria were found. We further investigated the *in vivo* role of flagellin in *B. pseudomallei* infection. In contrast to the work by Chua et al. [62] but in agreement with other studies [64], [65], we observed (Figure 10) that flagella are not required for *in vivo* virulence during intranasal or intraperitoneal infection of BALB/c mice, diabetic rats or Syrian hamsters.

A previous study established that the NLRC4 inflammasome can respond not only to bacterial flagellin but also to a conserved T3SS inner rod protein such as PrgJ from *S. typhimurium*, EprJ and EscI from *E. coli*, MxiI from *S. flexneri*, PscI from *P. aeruginosa* as well as BsaK from *B. pseudomallei* [11]. T3SS rod proteins including *S. typhimurium* PrgJ or *B. thailandensis* BsaK were shown to be specifically recognized by NAIP2 interacting with NLRC4 in mouse macrophages [13], [16]. Furthermore, the delivery of recombinant *B. thailandensis* BsaK into macrophages was able to induce NLRC4-dependent caspase-1 processing, pyroptosis and IL-1β maturation [13]. In line with these results, we found that deletion of *B. pseudomallei* BsaK abolishes caspase-1 activation and is associated with reduced LDH release and IL-1β secretion within 6 hours compared to the wild-type but at 24 hours no difference in caspase-1 activation, LDH release and IL-1β secretion between BsaK mutant and wild-type bacteria (Figures 9A, C, D) can be observed. Thus, we propose that BsaK is essential for early caspase-1-dependent macrophage death and IL-1β release, whereas other bacterial factors or mechanisms might contribute to caspase-1 activation in the late phase of infection. At this point, we cannot completely exclude that in addition to the absence of the BsaK protein, an impaired secretion of other caspase-1 activating proteins, or a potential delay in the vacuole escape of the BsaK mutant might also contribute to the missing caspase-1 activation in the early phase of infection. For *B. pseudomallei*, it has been suggested, that the NLRC4 inflammasome responds to T3SS3 deployment, which takes place early in the infection cycle, whereas activation of the NLRP3 inflammasome might require escape from the phagosome, which probably occurs later [12], [66]. In the present study we detected less LDH release (Figure 9C) and higher intracellular bacterial numbers (Figure 9B) in ΔBsaK compared to wild-type infected macrophages. In line with our previous work [29], we suggest that BsaK-induced caspase-1-dependent rapid macrophage death contributes to resistance by reducing the intracellular niche for *B. pseudomallei*, but in addition, caspase-1 might also have a role in controlling intracellular replication of the pathogen in macrophages. Thus, the T3SS3 inner rod protein BsaK seems to be important for induction of host defence against *B. pseudomallei in vitro*.

In contrast, we found a decreased mortality of BALB/c mice infected with the BsaK mutant compared to wild-type bacteria (Figure 10A), which might be due to a reduced secretion of the needle tip protein BipD (Figure 10C) or still unknown T3SS effectors. Disruption of the inner rod proteins of *Salmonella* (PrgJ) or *Yersinia* (YscI) has been shown to result in defective assembly of the needle complex [67]–[69] rendering bacteria incapable of secreting T3SS substrates [69]. Formation of the inner rod is thought to be critical for substrate specificity switching from the subunits of inner rod and needle proteins to the secretion of T3SS effectors of *Salmonella* [67], *Yersinia* [68], and *Escherichia* [70].

In the present study, challenge of BALB/c mice with *B. pseudomallei* BsaK mutant by the intranasal route resulted in reduced levels of IL-1β and neutrophil enzyme myeloperoxidase (Figure 11A), decreased influx of neutrophils (Figure 11B) and lower bacterial burden (Figure 10B). Previous work demonstrated that high amounts of IL-1β are deleterious during murine melioidosis because of excessive neutrophil influx into the lung and tissue damage [12]. As neutrophils do not express NLRC4 [18] and therefore do not undergo pyroptosis, they may be permissive to *B. pseudomallei* intracellular replication [12]. IL-1β might control recruitment of neutrophils to inflammatory sites by induction of chemokines such as neutrophil chemoattractants KC (CXCL1) and MIP-2 (CXCL2, CXCL3) or monocyte chemoattractant MCP-1 (CCL2). We found that secretion of MCP-1 is significantly decreased in BALF and serum (Figures 11A, S3) of ΔBsaK infected mice. Thus, ΔBsaK driven IL-1β-mediated reduced pulmonary neutrophil influx is possibly responsible for lower bacterial load in the lung and decreased dissemination of the pathogen (Figure 9B). In addition, IL-1β was reported to play a detrimental role during melioidosis by inhibiting activation of IFN-γ production [12], which was shown to be important for resistance against *B. pseudomallei*. We found that challenge of BALB/c mice with the BsaK mutant by the intravenous route resulted in increased IFN-γ production in serum (Figure S4). However, intranasal infection with ΔBsaK led to reduced IL-1β levels in BALF and IFN-γ levels in BALF and serum (Figures 11, S3). Thus, reduced IL-1β-mediated bacterial burden in lung of the pulmonary mouse model might be responsible for reduced inflammation overall.

In summary, we identified an important role of the *B. pseudomallei* T3SS3 inner rod protein BsaK in the early activation of NLRC4-dependent caspase-1 processing, pyroptosis and IL-1β secretion in macrophages and also in *B. pseudomallei in vivo* virulence. Our work also shows the activation of caspase-9 and -7 and cleavage of PARP downstream of NLRC4/caspase-1 inflammasome in the early phase of infection by *B. pseudomallei* and a possible role for caspase-1-dependent and -independent cell death mechanisms at later time points. Future studies will have to elucidate if differences in caspase activation and signalling among *B. pseudomallei* strains or *Burkholderia* spp. are linked to their virulence potential.

## Materials and Methods

### Ethics statement

All the animal experiments described in the present study were conducted in strict accordance with the recommendations in the Guide for the Care and Use of Laboratory Animals of the National Institutes of Health. All animal studies were conducted under a protocol approved by the Landesamt für Landwirtschaft, Lebensmittelsicherheit und Fischerei Mecklenburg-Vorpommern (LALLF M-V; 7221.3-1.1-020/11). All efforts were made to minimize suffering and ensure the highest ethical and human standards.

### Mice

Caspase-1/11-deficient and C57BL/6J wild-type mice were kindly provided by Arturo Zychlinski (Max Planck Institute for Infection Biology, Berlin, Germany). Caspase-7- deficient and C57BL/6J wild-type mice were purchased from The Jackson Laboratory (Bar Harbor, Maine, US). Breeding of mouse strains was performed by the Department of Laboratory Animal Science of the University Medicine of Greifswald (Greifswald, Germany) and MICROMUN (Greifswald, Germany), respectively. NLRC4-deficient and B6.129 wild-type mice as well as NLRP3-deficient and C57BL/6J wild-type mice were kindly provided by Markus Gerhard (Technical University of Munich, Germany). BALB/c mice were obtained from Charles River (Wiga Sulzfeld, Germany). Female mice (10–12 weeks) were housed in filter-top cages under standard conditions in physical containment level 3 laboratory facilities and provided with food and water ad libitum.

### Bacteria

*B. thailandensis* wild-type strain E264 (ATCC 700388) is an environmental isolate obtained from a rice field soil sample in central Thailand. *B. pseudomallei* wild-type strain E8 and strain E212 comprise soil isolates from the surrounding area of Ubon Ratchathani in northeast Thailand; *B. pseudomallei* strains K96243 and 1026b are clinical isolates from Thailand. Bacteria were grown on Columbia agar at 37°C for 24 hours and adjusted to the desired concentration in Dulbecco's phosphate-buffered saline (D-PBS; Life Technologies, Darmstadt, Germany) or the respective cell culture medium.

### Generation and cultivation of primary murine macrophages

Bone marrow-derived macrophages were generated and cultivated in a serum-free cell culture system as recently described [71]. Briefly, tibias and femurs were aseptically removed and bone marrow cells were flushed with sterile D-PBS and then centrifuged at 150× g for 15 min. Cells were resuspended in RPMI medium 1640 (Life Technologies) containing 5% “Panexin BMM” (PAN Biotech, Aidenbach, Germany), 2 ng/ml recombinant murine GM-CSF (PAN Biotech), and 50 µM mercaptoethanol and cultivated at least 10 days at 37°C in a humidified atmosphere containing 95% air and 5% CO_2_.

### Antibiotic protection assay

24 hours prior to infection, BMM were seeded in 48 well plates (1.2×10^5^ cells per well), and infected with *B. pseudomallei* strain E8, *B. pseudomallei* E8 mutants or *B. thailandensis* strain E264 at the indicated MOI. For invasion assays with the FliC mutant (ΔFliC) well plates were additionally centrifuged for 2 min at 120× g. After infection for 30 min cells were washed twice with D-PBS and incubated in 100 µg/ml kanamycin containing medium to eliminate remaining extracellular bacteria. To minimize re-infection and extracellular replication the culture medium was replaced by fresh medium containing 100 µg/ml kanamycin six hours after infection. At indicated time points (time zero was taken 25 min after incubation with antibiotic-containing medium) the number of intracellular colony forming units (CFU) was determined. Consequently, cells were washed twice with D-PBS and subsequently lysed using 150 µl D-PBS containing 0.5% Tergitol TMN (Fluka, Buchs, Switzerland) and 1% bovine serum albumin (BSA) per well. After 15 min of incubation appropriate dilutions of lysates were plated on Mueller-Hinton agar and incubated at 37°C for 48 hours.

### Lactate dehydrogenase assay

To quantify the extent of pyroptosis after bacterial infection, release of lactate dehydrogenase (LDH) in cell culture supernatants was determined. BMM were seeded in 48 well plates (1.2×10^5^ cells per well) and infected at the indicated MOI with *B. pseudomallei* strain E8, *B. pseudomallei* E8 mutants or *B. thailandensis* strain E264 for 30 min. Cells were washed twice with D-PBS, and 400 µl of medium containing 100 µg/ml of kanamycin was added to each well to eliminate extracellular bacteria. At the indicated time points, cell culture supernatant was collected, and LDH activity was detected by using the CytoTox-One homogeneous membrane integrity assay (Promega, Mannheim, Germany) according to the manufacturer's instructions. Briefly, 50 µl of supernatant was added to the kit reagent and incubated for 10 min at room temperature. After addition of stopping solution, the fluorescence intensity was measured using the microplate reader Infinite M200 PRO (Tecan, Crailsheim, Germany) at excitation wavelength of 560 nm and emission wavelength of 590 nm.

### Transposon mutagenesis of fliC

*B. pseudomallei* was mutagenized with Tn5-OT182 and examined for mutants exhibiting defects in plaque formation in a plaque-assay screen using PtK2 epithelial cells as previously described [32]. In one of the mutants that exhibited reduced plaque formation compared to the *B. pseudomallei* wild-type strain E8, the transposon insertion was identified in the BPSL3319 locus (fliC).

### Targeted mutagenesis of bsaU, bopE and bsaK

To construct markerless *B. pseudomallei* mutants, the vector pEXKm5 was used as previously described [72]. Up- and downstream PCR products of the BPSS1539 (bsaU) gene locus (Table S1) were fused together by overlap extension PCR and cloned into pEXKm5 resulting in the plasmid pEXKm5-ΔBPSS1539 (Table S2). Up- and downstream PCR products of the BPSS1525 (bopE) or BURPS1710b (bsaK) locus (Table S1) were digested with EcoRI and ligated with T4 ligase, and subsequently cloned into pEXKm5 resulting in the plasmid pEXKm5-ΔBPSS1525 and -ΔBURPS1710b, respectively (Table S2). Plasmids were introduced into *E. coli* RHO3 by heat shock transformation and selected on LB plates containing 37.5 µg/ml kanamycin and 400 µg/ml 2.6-diaminopimelic acid (DAP). For conjugation, *B. pseudomallei* E8 overnight cultures were mixed with pEXKm5-ΔBPSS1539, -ΔBPSS1525 or -ΔBURPS1710b containing *E. coli* RHO3 overnight cultures. Bacterial suspensions were washed and resuspended in 10 mM MgSO_4_ and delivered onto cellulose-acetate membrane filters on LB agar plates with 400 µg/ml DAP. *B. pseudomallei* colonies with chromosomally integrated pEXKm5 were identified on LB agar plates containing 1000 µg/ml kanamycin and 50 µg/ml 5-Brom-4-chlor-3-indolyl-β-D glucuronic acid (X-Gluc). Blue colonies were incubated at least 4 hours in YT broth (yeast extract/tryptone), plated onto YT agar plates containing 15% sucrose/50 µg/ml X-Gluc and incubated at 37°C for 48 hours. For further characterization white colonies were inoculated in LB broth with 50 µg/ml X-Gluc and chromosomal DNA was purified by phenol-chloroform-isoamylalcohol extraction method. Screening for bsaU, bopE or bsaK deletion was then performed via PCR.

### Plasmid construction of caspase-1 and bopE

Caspase-1 was amplified from murine macrophage cDNA and bopE from genomic DNA of the *B. pseudomallei* strain E8 or the *B. thailandensis* strain E264 (Table S1). PCR products were cloned into pcDNA-Flag (caspase-1) and pcDNA-Myc (bopE), respectively (Table S2).

### Mutagenesis of the bopE GEF domain

Mutation of the *B. pseudomallei* bopE GEF domain (bopE-R207E/N216P) was done by amino acid replacement using overlapping extension PCR as previously described [37]. For each mutation three PCRs were performed. At first two fragments were amplified with up- and downstream primers as well as complementary primers which contain the respective mutation (Table S1). The resulting DNA fragments were used as template for a third PCR to amplify the mutated gene. The product was purified and cloned into pcDNA-Myc (Table S2).

### Transient transfection of HEK293 cells

Human embryonic kidney 293 (HEK293) cells were seeded in 6 well plates (6.25×10^5^ cells per well, DMEM, 10% FCS) for 24 hours. 1.5 µg of pcDNA-caspase-1-Flag and 2 µg of respective pcDNA-bopE-Myc were cotransfected using Lipofectamine LTX (Life Technologies) according to the manufacturer's instructions. 24 hours after transfection cells were lysed with TRIzol Reagent (Life Technologies) and protein extraction was performed according to the manufacturer's protocol.

### Protein extraction and western blot analysis

24 hours prior to infection, BMM were seeded in 6 well plates (6.5×10^5^ cells per well), and infected for 30 min with *B. pseudomallei* strains (E8, K96243, E212, 1026b), *B. pseudomallei* E8 mutants or *B. thailandensis* strain E264 at the indicated MOI. Proteins of BMM were prepared by TRIzol Reagent according to manufacturer's instructions. Protein content was determined using the Bradford method. Equal amounts of protein were separated by SDS-PAGE and transferred onto nitrocellulose membranes by electroblotting. Membranes were blocked with 1× Roti-Block (Roth, Karlsruhe, Germany) for one hour at room temperature and subsequently incubated overnight at 4°C with a rabbit anti-GAPDH (AbFrontier, Acris Antibodies, Herford, Germany), rabbit anti-Caspase-1 (Santa Cruz Biotechnology, Heidelberg, Germany), rabbit anti-Caspase-3, rabbit anti-Caspase-7, rabbit anti-Caspase-8, rabbit anti-Caspase-9, rabbit-anti-PARP, rabbit anti-Phospho-JNK, and rabbit anti-Phospho-p38 MAPK (Cell Signaling Technology, Frankfurt am Main, Germany) antibody (in 20 mM Tris, 138 mM NaCl, pH 7.6, 5% (w/v) BSA, 0.1% (v/v) Tween 20). Horseradish peroxidase (HRP)-conjugated anti-rabbit IgG (in 1× Roti-Block) was used as a secondary antibody for one hour at room temperature. The LumiGLO system (Cell Signaling Technology) was used for detection. Nitrocellulose membranes were reused for detection of several target proteins.

Overnight *B. pseudomallei* cultures were adjusted in 50 ml LB to an OD_650_ of 0.01. Bacterial strains were grown in a shaking incubator for six hours. One tablet of Complete Mini Protease inhibitor cocktail (Roche Applied Science, Mannheim, Germany) was added per 50 ml culture and centrifuged for 10 min at 9.000× g. Supernatants were harvested and trichloroacetic acid was added to a final concentration of 10%. After incubation at 4°C overnight proteins were harvested by centrifugation at 15.000× g for 30 min. Proteins were washed 3 times with 98% ethanol and one time with 70% ethanol. Protein pellets were air dried, resuspended in 8 M urea/2 M thiourea and stored at −20°C until use. Equal amounts of protein were separated by SDS-PAGE and detected with monoclonal rabbit anti-BipD or anti-BopE antibodies (kindly provided by M. P. Stevens, University of Edinburgh, Scotland, UK) and HRP-conjugated anti-rabbit IgG.

### *In vivo* infection and determination of mortality and bacterial organ load

Experimental melioidosis was induced by either intranasal or intravenous inoculation of BALB/c mice with *B. pseudomallei* strain E8 or respective mutants. For intranasal infection, mice were anesthetized intraperitoneally by Ketamin/Rompun and inoculated with 30 µl of bacterial suspension (40 CFU in D-PBS) as recently described [73]. For intravenous infection mice received 200 µl of inoculum (250 CFU in D-PBS) into the lateral tail vein. Mice were monitored every 24 hours for survival after infection. To evaluate the bacterial burden of internal organs, mice were sacrificed 48 hours after infection, and their lungs, livers, and spleens were homogenized in D-PBS containing 0.5% Tergitol and 1% bovine serum albumin and plated onto Ashdown agar plates in appropriate dilutions. Data are presented as the total bacterial count (CFU) per organ.

### Serum and bronchoalveolar lavage fluid (BALF)

Blood was collected from the orbital sinus in anesthetized BALB/c mice 48 hours after infection. Animals were sacrificed and the tracheas were exposed through midline incision and cannulated with a sterile 20-gauge Introcan Safety intravenous catheter (Braun, Melsungen, Germany). The lungs were instilled and aspirated serially with 1 ml aliquots of sterile D-PBS for a total of 2 ml. BAL cells were pelleted by centrifugation and resuspended in autoMACS Buffer (Miltenyi Biotec, Bergisch Gladbach, Germany). The resulting supernatant (BALF) was stored at −70°C until use.

### Preparation of lung and spleen tissue

Lymphocytes from lung or spleen were prepared using modified protocols as previously described [74]–[76]. Lungs were excised and cut into 1–2 mm^2^ pieces, followed by a 60 min digestion at 37°C with collagenase/dispase (0.2 mg/ml each, Roche Applied Science) and DNase (25 µg/ml, Sigma-Aldrich, Taufkirchen, Germany) in D-PBS. To improve tissue decomposition lungs were pipetted every 10 min using a serologic pipette. EDTA was added to a final concentration of 5 mM followed by additional 5 min incubation at 37°C. Cells were passed through a 70 µm cell strainer and pelleted by centrifugation. Erythrocytes were lysed with 185 mM ammonium chloride solution containing 17 mM TRIS. Cells were pelleted by centrifugation and resuspended in autoMACS Buffer. Spleens were removed and splenocyte suspensions were produced by passing them through sterile 70 µm cell strainers. Cells were washed and resuspended in autoMACS Buffer.

### Flow cytometric analysis

Nonspecific antibody binding was blocked with FcR Blocking Reagent (Miltenyi Biotec). Antibodies used for cell-surface staining were CD45-APC-Cy7, F4/80-Pacific Blue (BioLegend, Fell, Germany), Gr-1-APC, CD11b-PerCPVio700, Siglec-f-PE, MHC-Class II-FITC, CD11c-FITC and CD49b-PE (Miltenyi Biotec). Cells were blocked, stained with antibodies according to the manufacturer's protocol, and fixed for 20 min in 2.25% formaldehyde [75]. Cells were washed with autoMACS Buffer and analysed using the MACSQuant Analyzer (Miltenyi Biotec). Gating strategy for identification of different subsets was based on Hackstein et al. [77]. Briefly, cells were gated based on scatter light (FSC, SSC) characteristics and viable singlet leukocytes were identified by CD45 expression. Out of the CD45-positive cells, neutrophils were identified by Gr-1^bright^ CD11b^bright^ expression. Out of the neutrophil negative fraction, eosinophils were identified by Siglec-f^+^ MHC-Class-II^neg^ expression. Out of the neutrophil and eosinophil negative fraction macrophages were identified as Siglec-f^++^ F4/80^+^ double positive cells. Total dendritic cells were identified as CD11c^+^ Siglec-f^neg^ CD49b^neg^ leukocytes. Cell numbers of subsets were calculated per organ.

### Measurement of cytokines and myeloperoxidase

Protein levels of cytokines (interleukin-6 (IL-6), monocyte chemoattractant protein-1 (MCP-1), interferon-γ (IFN-γ) and tumor necrosis factor-alpha (TNF-α) in serum and BALF were quantitatively measured using the Cytometric Bead Array (CBA) Mouse Inflammation Kit (BD Biosciences, Heidelberg, Germany) and flow cytometry with a MACSQuant Analyzer (Miltenyi Biotec) according to the manufacturer's protocols. Secretion of IL-1β was determined using a mouse IL-1β Single Analyte ELISArray Kit (SA Biosciences/QIAGEN, Hilden, Germany). Myeloperoxidase level in BALF was measured using a mouse MPO ELISA kit (Hycultec, Beutelsbach, Germany).

### Data presentation and statistical analysis

Figures and statistical analyses were performed using the GraphPad Prism 5.0. Comparisons between groups were conducted using Student's *t*-test or one-way ANOVA parametric test followed by the Dunnett or Bonferroni posthoc test for multiple comparisons as specified in the figure legends. The Dunnett test was used to compare infected groups with a single non-infected control group. Comparisons between infected groups were performed with the Bonferroni test. Survival curves were compared using the log-rank Kaplan-Meier test. *P* values of <0.05 were considered to be statistically significant.

## Supporting Information

Figure S1 **Early activation of caspase-9 in** ***Burkholderia*** **infected macrophages occurs upstream of caspase-7 and requires caspase-1.** Cleavage of caspases-1, -9, -7, and PARP was detected by immunoblot in cell lysates of caspase-9-inhibited (50 µM Ac-LEHD-CHO) C57BL/6 BMM infected with *B. pseudomallei* E8 and *B. thailandensis* E264 at MOI of 50∶1 at 1.5 hours post infection. One experiment of at least three performed is shown. non-infected (n.i.).
(TIF)

Figure S2 **NLRC4 is not required for late activation of caspase-1 in macrophages in response to** ***Burkholderia*** **.** Processing of caspase-1 and -7 was detected by immunoblot in cell lysates of NLRC4-deficient and B6.129 wild-type BMM infected with *B. pseudomallei* E8 and *B. thailandensis* E264 at MOI of 50∶1 at 12 hours post infection. One experiment of two performed is shown. non-infected (n.i.).
(TIF)

Figure S3 ***B. pseudomallei*** **ΔBsaK infected mice show reduced cytokine levels in serum.** BALB/c mice were intranasally infected with *B. pseudomallei* E8 wild-type and ΔBsaK at 40 CFU. Cytokine (TNF-α, IL-6, MCP-1, IFN-γ) levels were measured in serum obtained 48 hours after infection. Pooled data from two independent experiments are presented as mean with standard error of the mean (n = 10). Statistical analyses were performed using a Student's *t* test (**p<0.01; ***p<0.001).
(TIF)

Figure S4 ***B. pseudomallei*** **BsaK mutant is less virulent in a systemic mouse model.** BALB/c mice were intravenously infected with *B. pseudomallei* E8 wild-type and ΔBsaK at 250 CFU and sacrificed 48 hours after infection. (**A**) The bacterial load in lung, liver and spleen and (**B**) cytokines (IL-6, MCP-1, IFN-γ) in serum were determined. Pooled data from three independent experiments are presented as mean (n = 8–9). Statistical analyses were performed using a Student's *t* test (*p<0.05; **p<0.01; ***p<0.001).
(TIF)

Figure S5 **Activation of caspase-7 downstream of caspase-1 does not restrict** ***Burkholderia*** **replication in macrophages.** (**A**) Secretion of IL-1β was determined in cell culture supernatants of BMM from caspase-7-deficient and C57BL/6 wild-type mice 12 hours after infection with *B. pseudomallei* E8 or *B. thailandensis* E264 (MOI 50∶1). (**B**) Invasion and intracellular bacterial growth of *B. pseudomallei* E8 and *B. thailandensis* E264 was examined in respective BMM infected at MOI of 2∶1 at the indicated time points. (**C**) Induction of cytotoxicity was measured as lactate dehydrogenase (LDH) release in supernatants of *Burkholderia* infected caspase-7-deficient and wild-type BMM (MOI 2∶1 or 200∶1). (A) Data are presented as mean with standard error of the mean (SEM) of four independent experiments (n = 4). (B, C) Data are presented as mean with SEM of triplicate determinations. One representative experiment out of three independent experiments is shown. Statistical analyses were performed using one-way ANOVA (*p<0.05; **p<0.01; ***p<0.001 compared to non-infected macrophages or as indicated). below detection (bd), non-infected (n.i.).
(TIF)

Figure S6 **Caspase-7 does not play an important role in resistance in murine melioidosis.** (**A**) Caspase-7-deficient and C57BL/6 wild-type mice mice were intranasally infected with *B. pseudomallei* E8 at 500 CFU and survival was monitored (log rank Kaplan-Meier test). Pooled data from two independent experiments are shown (n = 9). (**B**) Mice were sacrificed 48 hours after infection and the bacterial load (CFU) in lung, liver and spleen was determined. (**C**) Cytokine (TNF-α, IL-6, MCP-1, IFN-γ) levels were measured in serum obtained 48 hours after infection. (B, C) Pooled data from two independent experiments are presented as mean with standard error of the mean (n = 9). Statistical analyses were performed using a Student's *t* test (*p<0.05).
(TIF)

Figure S7 ***Burkholderia*** **mediates pyroptosis via the host receptor NLRC4 but not NLRP3.** (**A, B**) Invasion and intracellular bacterial growth of *B. pseudomallei* E8 and *B. thailandensis* E264 was examined in BMM from (A, left) NLRC4-deficient, (B, left) NLRP3- deficient and respective wild-type BMM infected at MOI of 2∶1. Induction of cytotoxicity was measured as lactate dehydrogenase (LDH) release in cell culture supernatants of *B. pseudomallei* E8 or *B. thailandensis* E264 infected (A, right) NLRC4-deficient or (B, right) NLRP3-deficient BMM (MOI 200∶1). (A, B) Data are presented as mean with standard error of the mean (SEM) of triplicate determinations. One representative experiment out of two independent experiments is shown. Statistical analyses were performed using one-way ANOVA (*p<0.05; **p<0.01; ***p<0.001 compared to non-infected macrophages or as indicated). non-infected (n.i.).
(TIF)

Figure S8 ***Burkholderia*** **infection of macrophages does not result in cleavage of caspase-11.** (**A**) Expression of caspase-11 was analysed by immunoblot in cell lysates of caspase-1/11-deficient and C57BL/6 wild-type BMM infected with *B. pseudomallei* E8 and *B. thailandensis* E264 at MOI of 50∶1 at 1.5 hours post infection. (**B**) Cleavage of caspases-11, -1, -9, -7, and PARP was analysed by immunoblot in cell lysates of C57BL/6 BMM infected with *B. pseudomallei* strain E8 and *B. thailandensis* strain E264 at MOI of 2∶1 or 50∶1 at 1.5 hours post infection. One experiment of at least three performed is shown. non-infected (n.i.).
(TIF)

Figure S9 ***B. pseudomallei*** **ΔBsaK infected mice show reduced levels of dendritic cells.** BALB/c mice were intranasally infected with *B. pseudomallei* E8 wild-type and ΔBsaK at 40 CFU. Flow cytometric analyses for dendritic cells in BALF, lung and spleen was performed 48 hours after challenge with *B. pseudomallei* E8 wild-type, ΔBsaK or PBS. Pooled data from two independent experiments are presented as mean with standard error of the mean (n = 10). Statistical analyses were performed using one-way ANOVA (*p<0.05 compared to PBS infected mice or as indicated).
(TIF)

Table S1 **PCR primers used for plasmid construction.**
(DOC)

Table S2 **Plasmids and bacterial strains used in this study.**
(DOC)
