# Supplementary figures and images for: Caspase-1-Dependent and -Independent Cell Death Pathways in Burkholderia pseudomallei Infection of Macrophages
Source: PLoS Pathog. 2014 Mar 13;10(3):e1003986. doi: 10.1371/journal.ppat.1003986 (PMC3953413; doi:10.1371/journal.ppat.1003986)

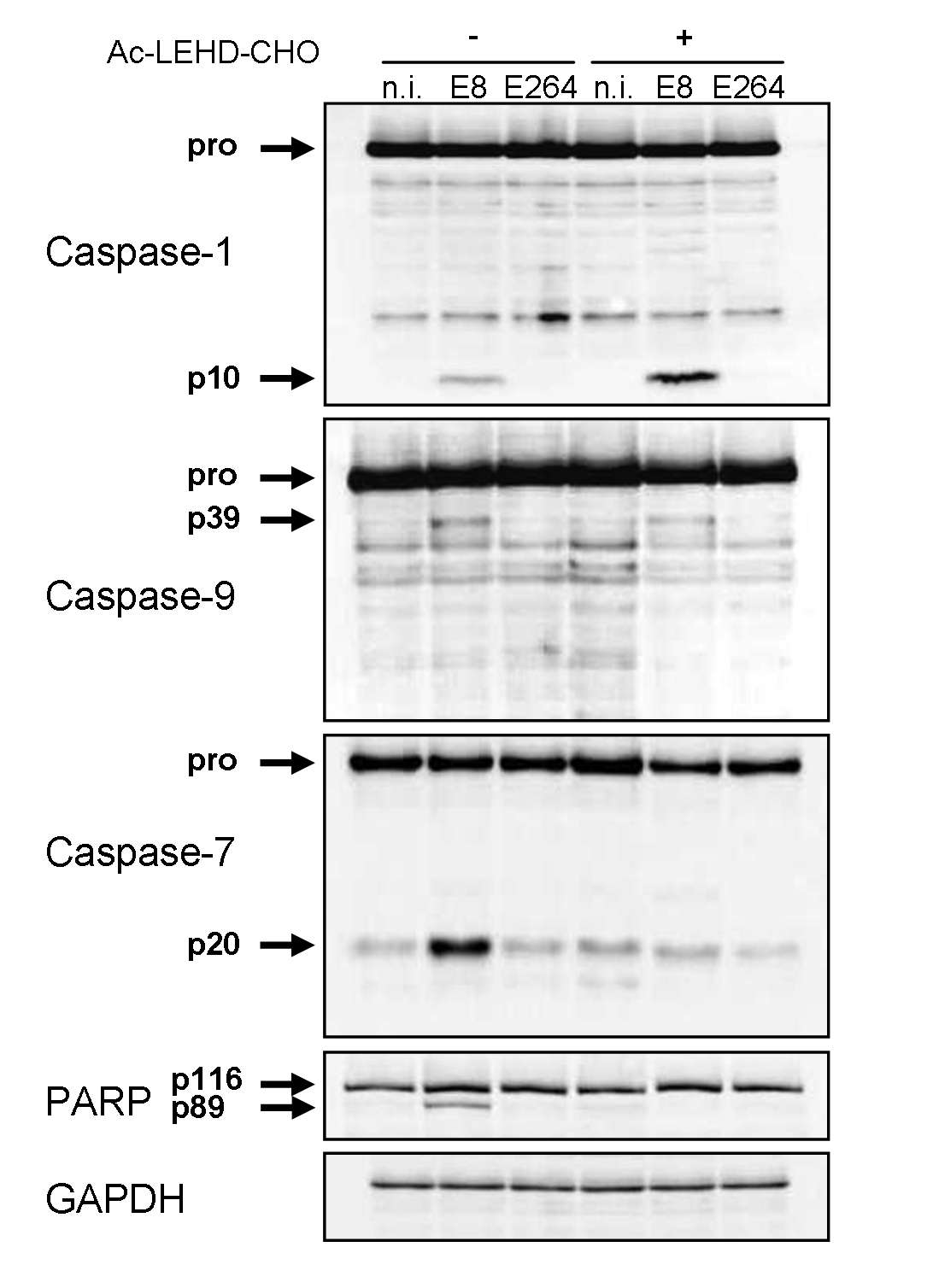

Supplement: Figure S1 — Early activation of caspase-9 in Burkholderia infected macrophages occurs upstream of caspase-7 and requires caspase-1. Cleavage of caspases-1, -9, -7, and PARP was detected by immunoblot in cell lysates of caspase-9-inhibited (50 µM Ac-LEHD-CHO) C57BL/6 BMM infected with B. pseudomallei E8 and B. thailandensis E264 at MOI of 50∶1 at 1.5 hours post infection. One experiment of at least three performed is shown. non-infected (n.i.). (TIF) [file ppat.1003986.s001.tif]

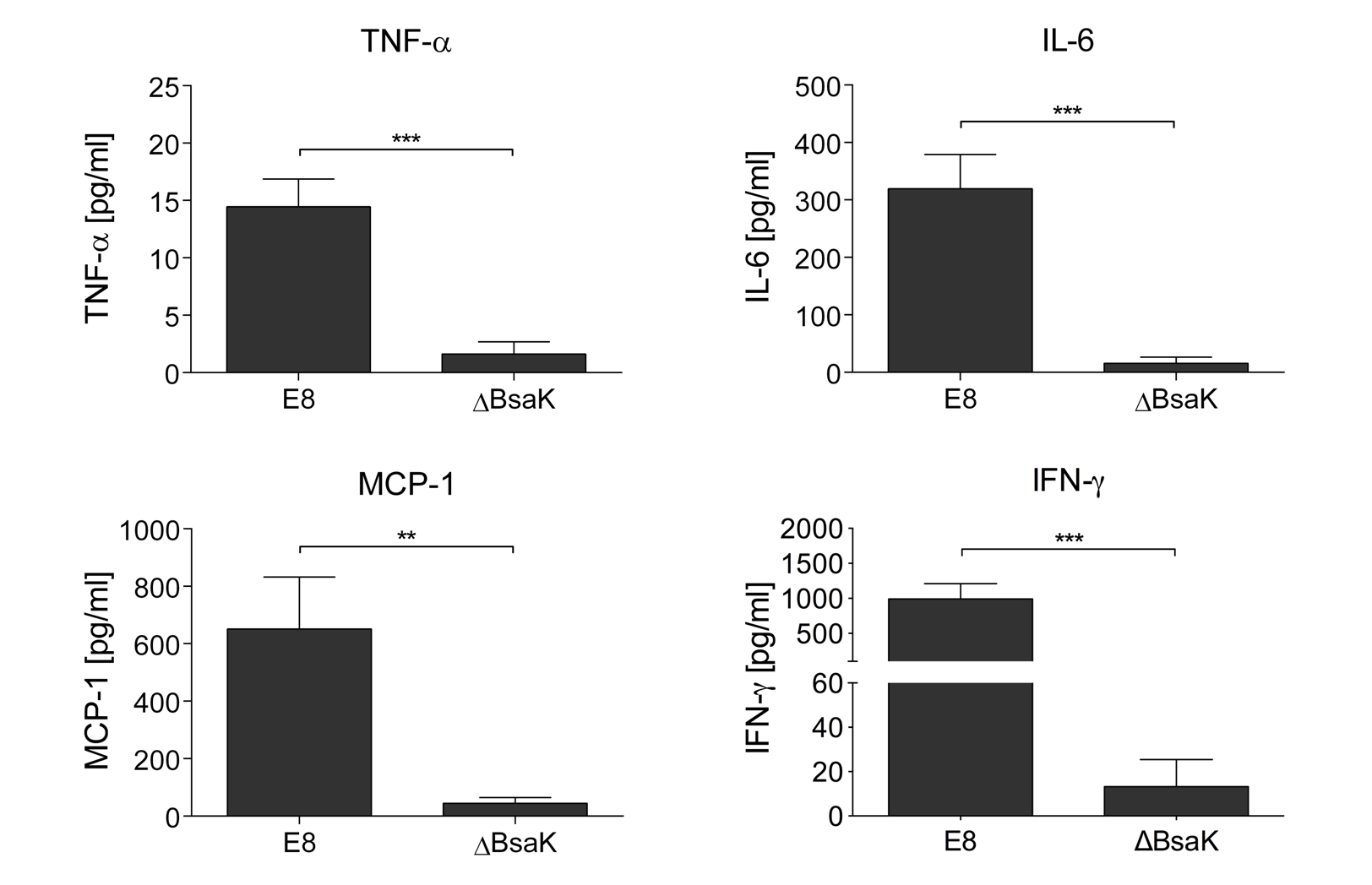

Supplement: Figure S3 — B. pseudomallei ΔBsaK infected mice show reduced cytokine levels in serum. BALB/c mice were intranasally infected with B. pseudomallei E8 wild-type and ΔBsaK at 40 CFU. Cytokine (TNF-α, IL-6, MCP-1, IFN-γ) levels were measured in serum obtained 48 hours after infection. Pooled data from two independent experiments are presented as mean with standard error of the mean (n = 10). Statistical analyses were performed using a Student's t test (**p<0.01; ***p<0.001). (TIF) [file ppat.1003986.s003.tif]

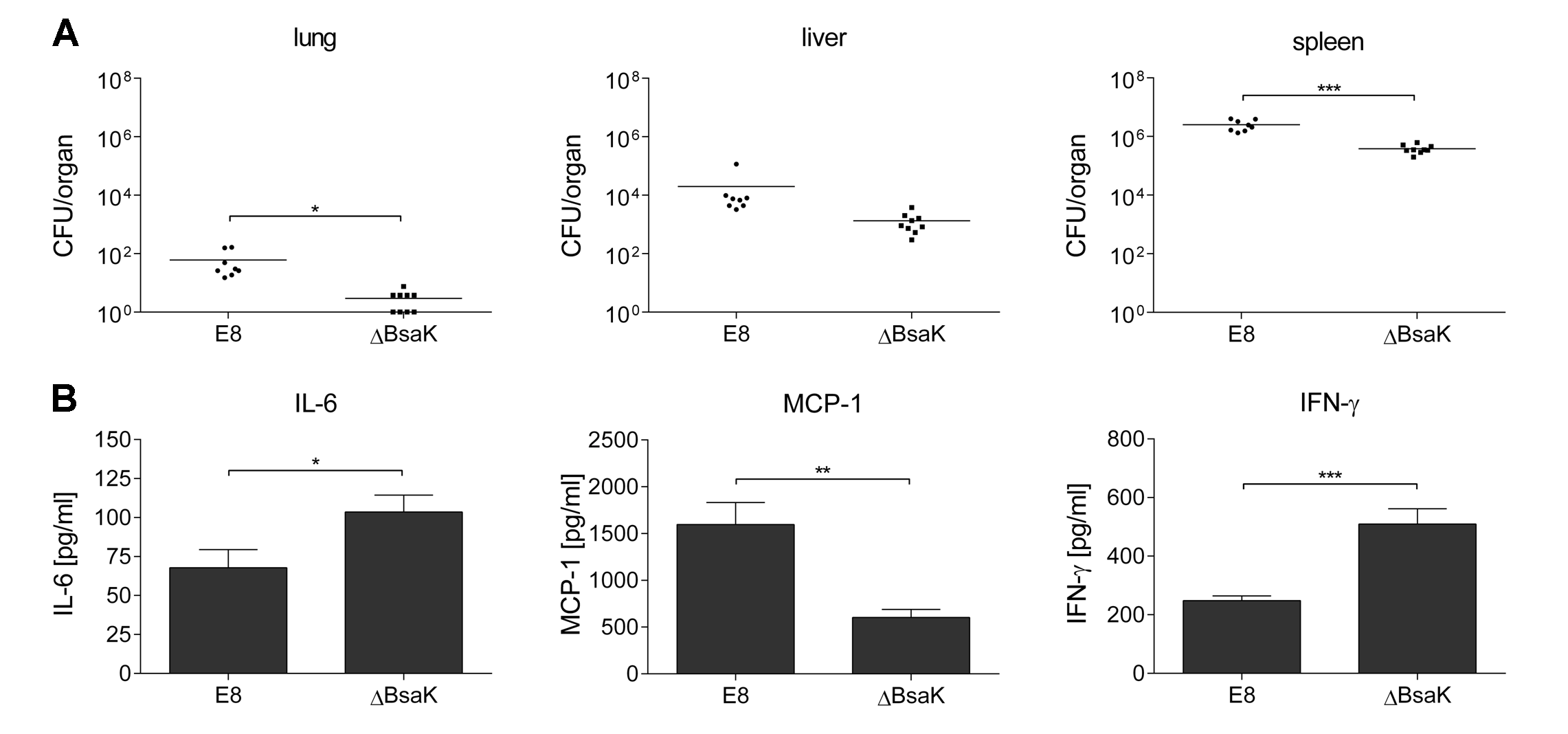

Supplement: Figure S4 — B. pseudomallei BsaK mutant is less virulent in a systemic mouse model. BALB/c mice were intravenously infected with B. pseudomallei E8 wild-type and ΔBsaK at 250 CFU and sacrificed 48 hours after infection. (A) The bacterial load in lung, liver and spleen and (B) cytokines (IL-6, MCP-1, IFN-γ) in serum were determined. Pooled data from three independent experiments are presented as mean (n = 8–9). Statistical analyses were performed using a Student's t test (*p<0.05; **p<0.01; ***p<0.001). (TIF) [file ppat.1003986.s004.tif]

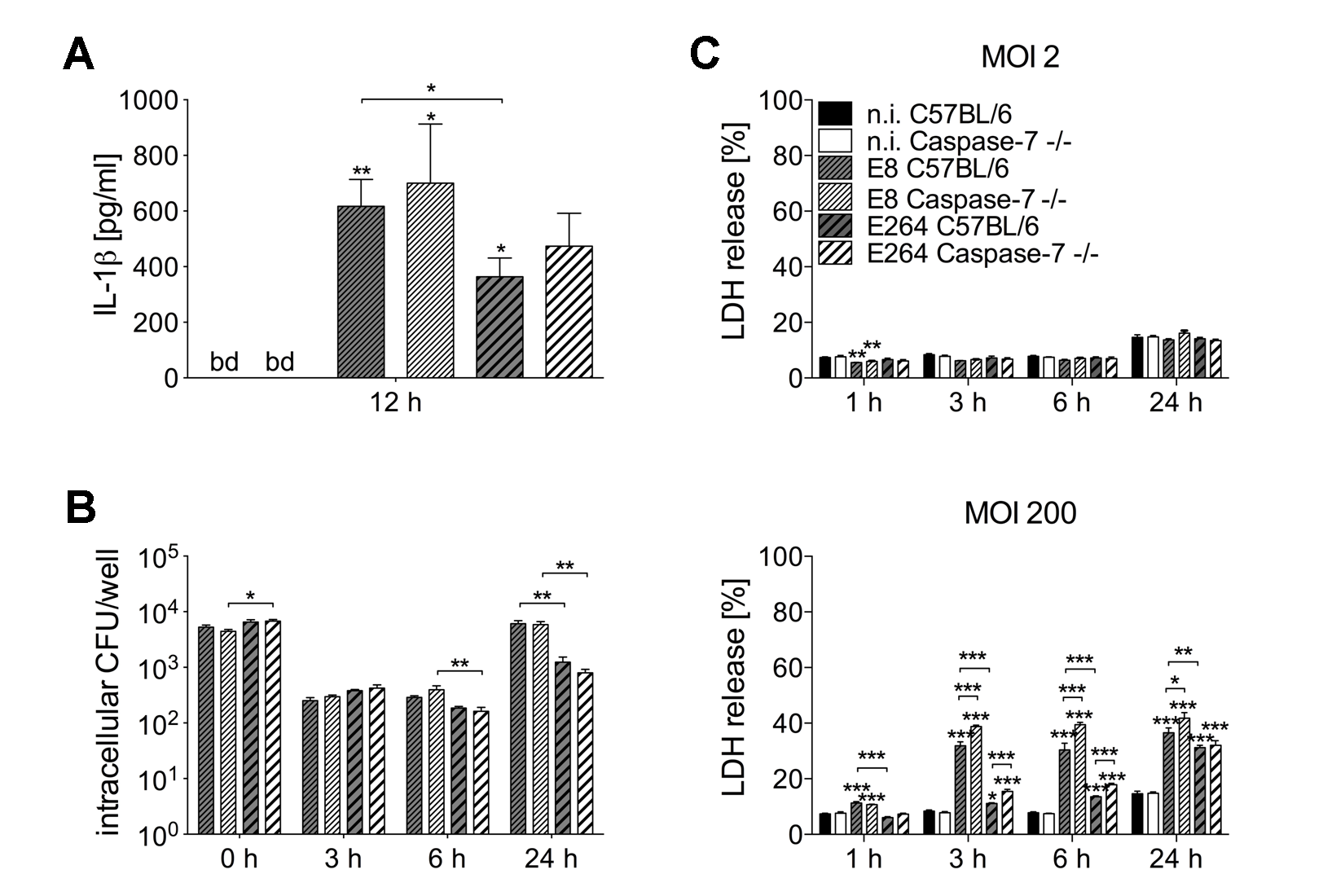

Supplement: Figure S5 — Activation of caspase-7 downstream of caspase-1 does not restrict Burkholderia replication in macrophages. (A) Secretion of IL-1β was determined in cell culture supernatants of BMM from caspase-7-deficient and C57BL/6 wild-type mice 12 hours after infection with B. pseudomallei E8 or B. thailandensis E264 (MOI 50∶1). (B) Invasion and intracellular bacterial growth of B. pseudomallei E8 and B. thailandensis E264 was examined in respective BMM infected at MOI of 2∶1 at the indicated time points. (C) Induction of cytotoxicity was measured as lactate dehydrogenase (LDH) release in supernatants of Burkholderia infected caspase-7-deficient and wild-type BMM (MOI 2∶1 or 200∶1). (A) Data are presented as mean with standard error of the mean (SEM) of four independent experiments (n = 4). (B, C) Data are presented as mean with SEM of triplicate determinations. One representative experiment out of three independent experiments is shown. Statistical analyses were performed using one-way ANOVA (*p<0.05; **p<0.01; ***p<0.001 compared to non-infected macrophages or as indicated). below detection (bd), non-infected (n.i.). (TIF) [file ppat.1003986.s005.tif]

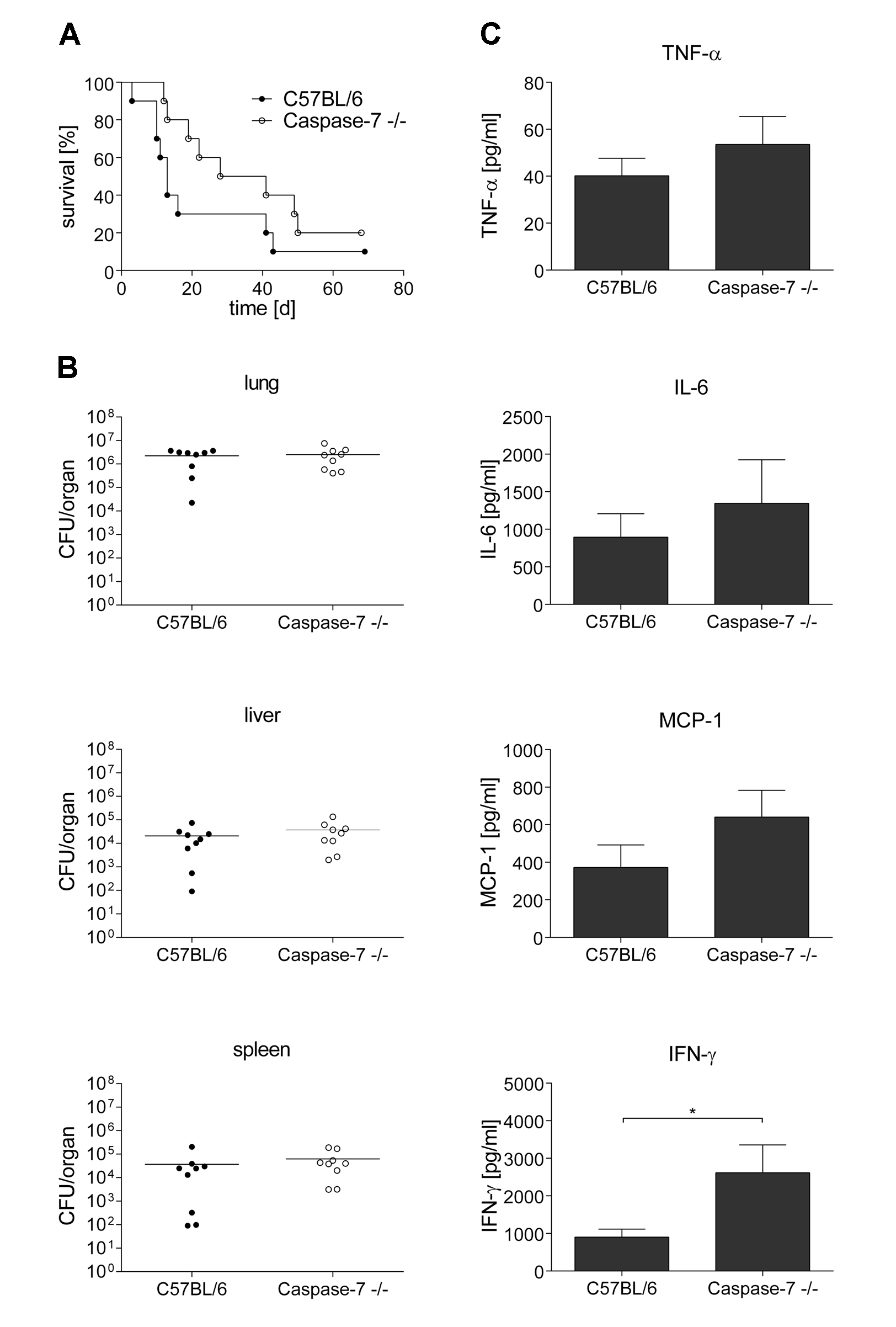

Supplement: Figure S6 — Caspase-7 does not play an important role in resistance in murine melioidosis. (A) Caspase-7-deficient and C57BL/6 wild-type mice mice were intranasally infected with B. pseudomallei E8 at 500 CFU and survival was monitored (log rank Kaplan-Meier test). Pooled data from two independent experiments are shown (n = 9). (B) Mice were sacrificed 48 hours after infection and the bacterial load (CFU) in lung, liver and spleen was determined. (C) Cytokine (TNF-α, IL-6, MCP-1, IFN-γ) levels were measured in serum obtained 48 hours after infection. (B, C) Pooled data from two independent experiments are presented as mean with standard error of the mean (n = 9). Statistical analyses were performed using a Student's t test (*p<0.05). (TIF) [file ppat.1003986.s006.tif]

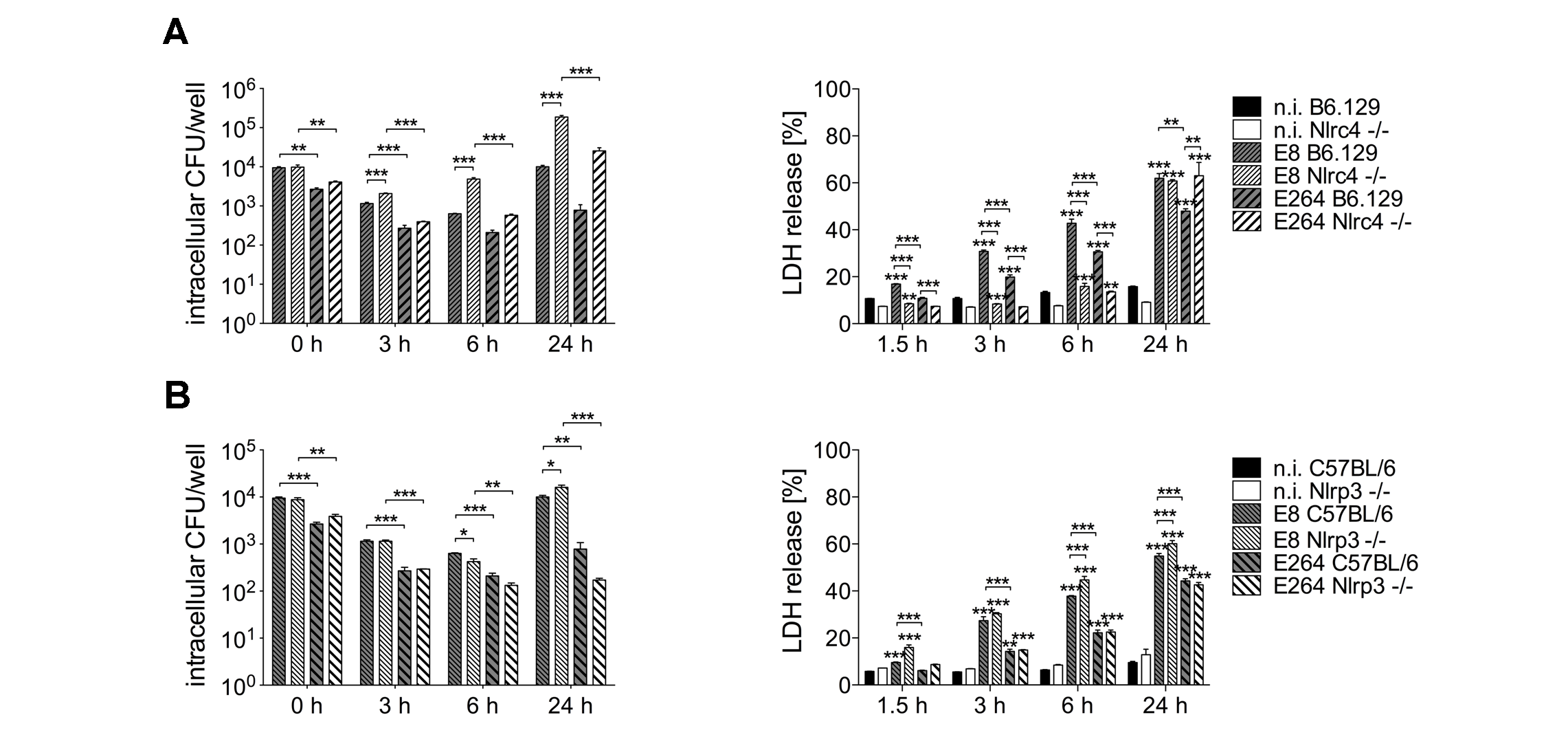

Supplement: Figure S7 — Burkholderia mediates pyroptosis via the host receptor NLRC4 but not NLRP3. (A, B) Invasion and intracellular bacterial growth of B. pseudomallei E8 and B. thailandensis E264 was examined in BMM from (A, left) NLRC4-deficient, (B, left) NLRP3- deficient and respective wild-type BMM infected at MOI of 2∶1. Induction of cytotoxicity was measured as lactate dehydrogenase (LDH) release in cell culture supernatants of B. pseudomallei E8 or B. thailandensis E264 infected (A, right) NLRC4-deficient or (B, right) NLRP3-deficient BMM (MOI 200∶1). (A, B) Data are presented as mean with standard error of the mean (SEM) of triplicate determinations. One representative experiment out of two independent experiments is shown. Statistical analyses were performed using one-way ANOVA (*p<0.05; **p<0.01; ***p<0.001 compared to non-infected macrophages or as indicated). non-infected (n.i.). (TIF) [file ppat.1003986.s007.tif]

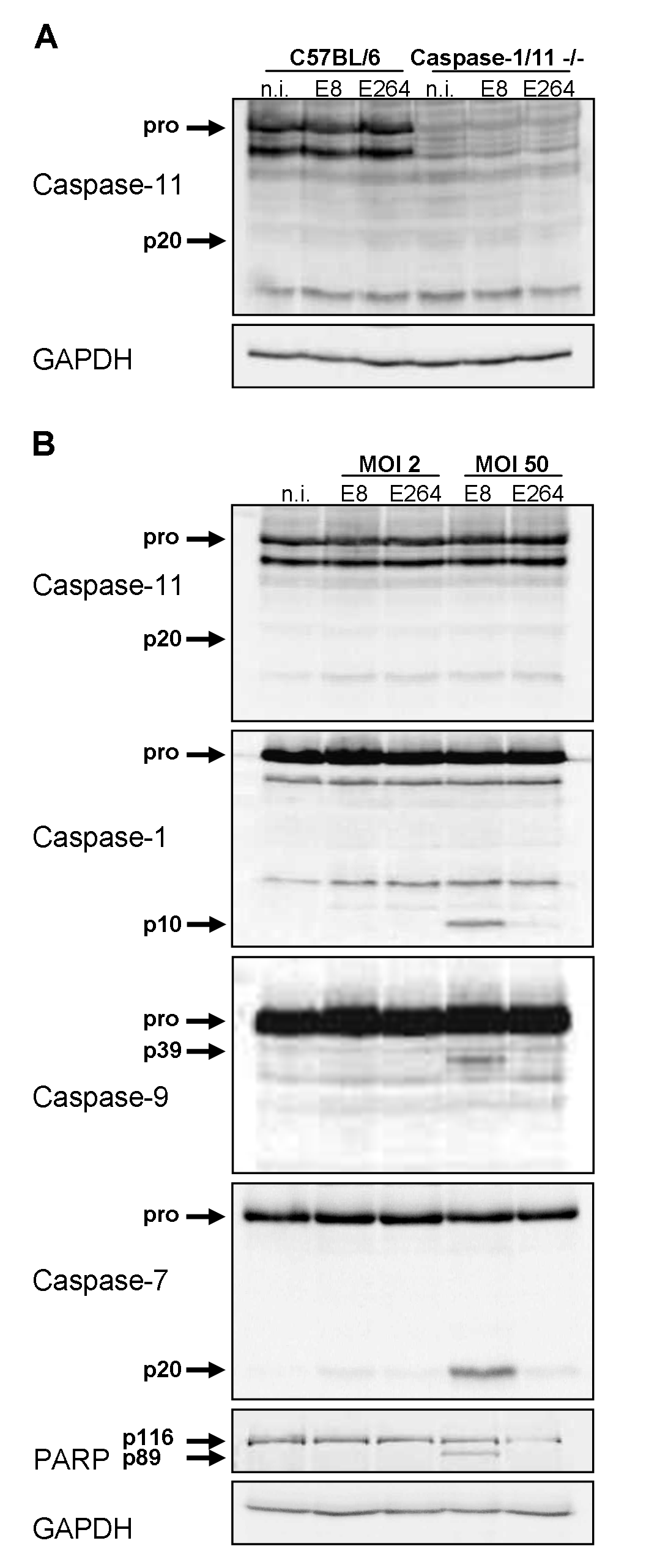

Supplement: Figure S8 — Burkholderia infection of macrophages does not result in cleavage of caspase-11. (A) Expression of caspase-11 was analysed by immunoblot in cell lysates of caspase-1/11-deficient and C57BL/6 wild-type BMM infected with B. pseudomallei E8 and B. thailandensis E264 at MOI of 50∶1 at 1.5 hours post infection. (B) Cleavage of caspases-11, -1, -9, -7, and PARP was analysed by immunoblot in cell lysates of C57BL/6 BMM infected with B. pseudomallei strain E8 and B. thailandensis strain E264 at MOI of 2∶1 or 50∶1 at 1.5 hours post infection. One experiment of at least three performed is shown. non-infected (n.i.). (TIF) [file ppat.1003986.s008.tif]

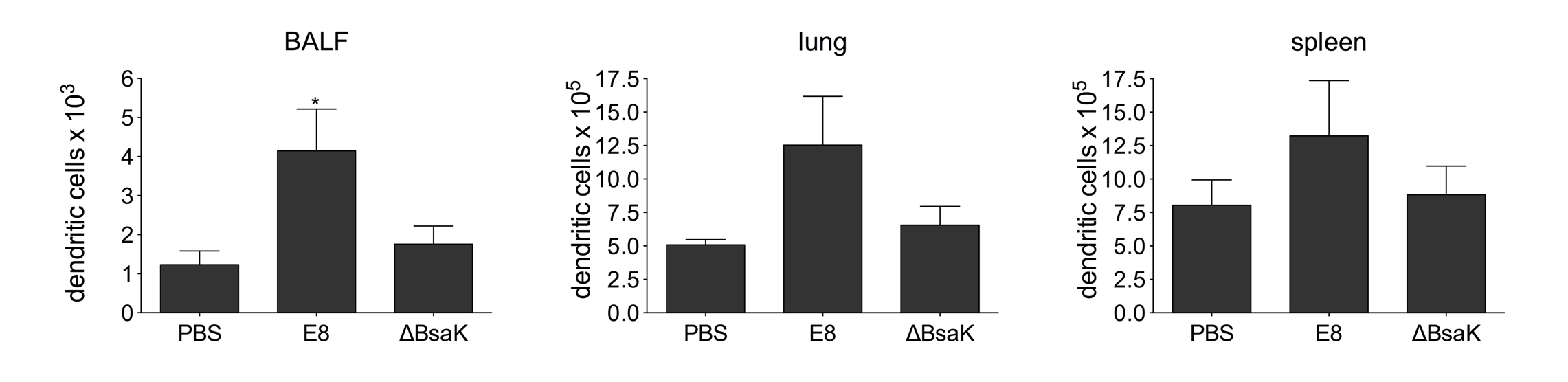

Supplement: Figure S9 — B. pseudomallei ΔBsaK infected mice show reduced levels of dendritic cells. BALB/c mice were intranasally infected with B. pseudomallei E8 wild-type and ΔBsaK at 40 CFU. Flow cytometric analyses for dendritic cells in BALF, lung and spleen was performed 48 hours after challenge with B. pseudomallei E8 wild-type, ΔBsaK or PBS. Pooled data from two independent experiments are presented as mean with standard error of the mean (n = 10). Statistical analyses were performed using one-way ANOVA (*p<0.05 compared to PBS infected mice or as indicated). (TIF) [file ppat.1003986.s009.tif]
