# Supplementary material for: Caspase-1-Dependent and -Independent Cell Death Pathways in Burkholderia pseudomallei Infection of Macrophages
Source: PLoS Pathog. 2014 Mar 13;10(3):e1003986. doi: 10.1371/journal.ppat.1003986 (PMC3953413; doi:10.1371/journal.ppat.1003986)
Supplement: Table S1 — PCR primers used for plasmid construction. (DOC) [file ppat.1003986.s010.doc]

**Table S1** PCR primers used for plasmid construction

| Primer name | Sequence 5’ → 3’ | PCR amplification |
| --- | --- | --- |
| **Transfection** |  |  |
| mCasp-1-for | GGATCCAACGCCATGGCTGACAAGAT | template: murine BMM |
| mCasp-1-rev | GCGGCCGCTCTTCGTTTAATGTCCCGGGA |  |
| Bp-bopE-for | GGATCCGACATGACTTACAACCCGAG | template: genomic DNA Bp E8 |
| Bp-bopE-rev | GCGGCCGCACCACGCTACCGCGTCAC |  |
| Bt-bopE-for | GGATCCCGAGAGCAAGACATGACTTA | template: genomic DNA Bt E264 |
| Bt-bopE-rev | CCGCGGGCTACTTCGTCATGCGCC |  |
| bopE-216-for-PCR-1 | CGACGTCGATGCTGAAGACATTC | template: genomic DNA Bp E8 |
| bopE-216-rev-PCR-1 | CAACGCGGGCGCGACGAGATC |  |
| bopE-216-for-PCR-2 | CTCGTCGCGCCCGCGTTGC | template: genomic DNA Bp E8 |
| bopE-216-rev-PCR-2 | GCTTGAGCGAGCGCTATGC |  |
| bopE-216-for-PCR-3 | CGACGTCGATGCTGAAGACATTC | template: products of PCR1 and PCR2 |
| bopE-216-rev-PCR-3 | GCTTGAGCGAGCGCTATGC |  |
| bopE-207-for-PCR-1 | CGACGTCGATGCTGAAGACATTC | template : bopE-216-PCR-3 |
| bopE-207-rev-PCR-1 | GCCTGCTGCTCCGCATAC |  |
| bopE-207-for-PCR-2 | GTATGCGGAGCAGCAGGC | template : bopE-216-PCR-3 |
| bopE-207-rev-PCR-2 | ACCACGCTACCGCGTCAC |  |
| bopE-207-for-PCR-3-BamHI | GGATCCGACATGACTTACAACCCGAG | template: products of PCR1 and PCR2 |
| bopE-207-rev-PCR-3-NotI | GCGGCCGCACCACGCTACCGCGTCAC |  |
| **Mutagenesis** |  |  |
| BPSS1539up_for | TCGACGGCCACGTGTATCTG | forward primer for *bsaU* upstream fragment |
| BPSS1539up_rev | GCGACGCGCCTGAACAACGTATAGCGCGGAACCCATGACCTGCTCCTTCC | reverse primer for *bsaU* upstream fragment |
| BPSS1539dn_for | ACATCACGGAAGGAGCAGGTCATGGGTTCCGCGCTATACGTTGTTCAGGC | forward primer for *bsaU* downstream fragment |
| BPSS1539dn_rev | TCGTGCAGCACGAACTCCAG | reverse primer for *bsaU* downstream fragment |
| BPSS1539ko-seq_for | GTTCTACACGGTGCTGCTCG | primer 832 bp upstream of *bsaU* orf |
| BPSS1539ko-seq_rev | TTCACCTCGACCGCGAGCTG | primer 925 bp downstream of *bsaU* orf |
| BPSS1525up_for | GTGCCGTGGAAGTGCTCG | forward primer for *bopE* upstream fragment |
| BPSS1525up_rev SacI | GAGCTCGTCTTGCTCTCGGTTGAAGG | reverse primer for *bopE* upstream fragment |
| BPSS1525dn_for SacI | GAGCTCGGATCCGACGGATTCGACG | forward primer for *bopE* downstream fragment |
| BPSS1525dn_rev | GATTCGGGAGGCGCATGAG | reverse primer for *bopE* downstream fragment |
| BPSS1525ko-seq_for | GAATTCCTTCGACAGCGCGAGCAC | primer 869 bp upstream of *bopE* orf |
| BPSS1525ko-seq_rev | GAATTCTGCATCGACGACGCCGCA | primer 900 bp downstream of *bopE* orf |
| BURPS1710b_A0590up_for | CTCAGGCGTCAGCGCGAG | forward primer for *bsaK* upstream fragment |
| BURPS1710b_A0590up_rev BamHI | GGATCCACTCGTAGTCGGCCAGCAG | reverse primer for *bsaK* upstream fragment |
| BURPS1710b_A0590dn_for BamHI | GGATCCCGCGATGAAGCGATTCGTCT | forward primer for *bsaK* downstream fragment |
| BURPS1710b_A0590dn_rev | CGGATGCATCGAAGGCGTC | reverse primer for *bsaK* downstream fragment |
| BURPS1710b_A0590ko-seq_for EcoRI | GAATTCGCGTCAGCGCGAGCCATG | primer 931 bp upstream of *bsaK* orf |
| BURPS1710b_A0590ko-seq_rev EcoRI | GAATTCCGGATATGTCGAATGCGTCG | primer 833 bp downstream of *bsaK* orf |
