# Supplementary material for: Caspase-1-Dependent and -Independent Cell Death Pathways in Burkholderia pseudomallei Infection of Macrophages
Source: PLoS Pathog. 2014 Mar 13;10(3):e1003986. doi: 10.1371/journal.ppat.1003986 (PMC3953413; doi:10.1371/journal.ppat.1003986)
Supplement: Table S2 — Plasmids and bacterial strains used in this study. (DOC) [file ppat.1003986.s011.doc]

**Table S2** Plasmids and bacterial strains used in this study

| Plasmid or strain | Relevant characteristics | Source / reference |
| --- | --- | --- |
| **Plasmids** |  |  |
| pcDNA-Flag | modified pcDNA3 vector containing flag tag | K. Breitbach |
| pcDNA-Flag-mCasp-1 | pcDNA-Flag containing 1209 bp murine caspase-1 orf | This study |
| pcDNA-Myc | modified pcDNA3 vector containing myc tag | K. Breitbach |
| pcDNA-Myc-Bp-bopE | pcDNA-Myc containing 786 bp *B. ps.* bopE orf | This study |
| pcDNA-Myc-Bp-bopE-R207E/N216P | pcDNA-Myc containing 786 bp *B. ps.* bopE R207E/N216P orf | This study |
| pcDNA-Myc-Bt-bopE | pcDNA-Myc containing 786 bp *B. th.* bopE orf | This study |
| pEXKm5 | Km^r^ , *gusA* reporter gene; *Bp* optimized *sacB* gene | Lopez et al., 2009 |
| pEXKm5-ΔBPSS1539 (BsaU) | pEXKm5 containing a 765 bp fragment upstream and 766 bp fragment downstream of the orf BPSS1539 | This study |
| pEXKm5-ΔBPSS1525 (BopE) | pEXKm5 containing a 869 bp fragment upstream and 781 bp fragment downstream of the orf BPSS1525 | This study |
| pEXKm5-ΔBURPS1710b_A0590 (BsaK) | pEXKm5 containing a 931bp fragment upstream and 833bp fragment downstream of the orf BURPS1710b_A0590 | This study |
| ***E. coli* strains** |  |  |
| XL1blue | recA1 endA1 gyrA96 thi-1 hsdR17 supE44 relA1 lac [F’ proAB lacIqZ_M15 Tn10 (Tetr)] | Stratagene |
| RHO3 | SM10(λpir)*Δasd::FRTΔaphA::FRT;* Km^s^; DAP auxotroph | Lopez et al. 2009 |
| ***B. pseudomallei* strains** |  |  |
| BpΔBPSS1539 (ΔBsaU) | E8 derivate; BPSS1539 (*bsaU*) orf was deleted | This study |
| BpΔBPSS1525 (ΔBopE) | E8 derivate; BPSS1525 (*bopE*) orf was deleted | This study |
| BpΔBURPS1710b_A0590 (ΔBsaK) | E8 derivate; BURPS1710b_A0590 (*bsaK*) orf was deleted | This study |
| BpΔBPSL3319 (ΔFliC) | E8 derivate, BPSL3319 (*fliC*) orf was destroyed by transposon | K. Eske-Pogodda |
| BpΔBPSL3319 + (ΔFliCΔBopE) | BpΔBPSL3319 derivate, BPSS1525 (*bopE*) was deleted | This study |
| BpΔBPSL3319 + (ΔFliCΔBsaK) | BpΔBPSL3319 derivate, BURPS1710b_A0590 (*bsaK*) was deleted | This study |

Abbreviations: Km^r^ = kanamycin resistant; Ap^r^ = ampicillin resistant; Zeo^r^ = zeocine resistant; DAP = 2,6-diaminopimelic acid.
